# Despite the odds: formation of the SARS-CoV-2 methylation complex

**DOI:** 10.1093/nar/gkae165

**Published:** 2024-03-18

**Authors:** Alex Matsuda, Jacek Plewka, Michał Rawski, André Mourão, Weronika Zajko, Till Siebenmorgen, Leanid Kresik, Kinga Lis, Alisha N Jones, Magdalena Pachota, Abdulkarim Karim, Kinga Hartman, Shivlee Nirwal, Ravi Sonani, Yuliya Chykunova, Igor Minia, Paweł Mak, Markus Landthaler, Marcin Nowotny, Grzegorz Dubin, Michael Sattler, Piotr Suder, Grzegorz M Popowicz, Krzysztof Pyrć, Anna Czarna

**Affiliations:** Virogenetics Laboratory of Virology, Malopolska Centre of Biotechnology, Jagiellonian University, 30-387 Kraków, Poland; Doctoral School of Exact and Natural Sciences, Jagiellonian University, 30-387 Kraków, Poland; Virogenetics Laboratory of Virology, Malopolska Centre of Biotechnology, Jagiellonian University, 30-387 Kraków, Poland; Faculty of Chemistry, Jagiellonian University, 30-387 Kraków, Poland; SOLARIS National Synchrotron Radiation Centre, Jagiellonian University, 30-392 Kraków, Poland; Helmholtz Zentrum München, 85764 Neuherberg, Germany; Laboratory of Protein Structure, International Institute of Molecular and Cell Biology, 02-109 Warsaw, Poland; Helmholtz Zentrum München, 85764 Neuherberg, Germany; Virogenetics Laboratory of Virology, Malopolska Centre of Biotechnology, Jagiellonian University, 30-387 Kraków, Poland; Virogenetics Laboratory of Virology, Malopolska Centre of Biotechnology, Jagiellonian University, 30-387 Kraków, Poland; Faculty of Chemical Engineering and Technology, Kraków University of Technology, 31-155 Kraków, Poland; Helmholtz Zentrum München, 85764 Neuherberg, Germany; Bavarian NMR Center, Department of Chemistry, Technical University of Munich, 85748 Garching, Germany; Virogenetics Laboratory of Virology, Malopolska Centre of Biotechnology, Jagiellonian University, 30-387 Kraków, Poland; Department of Microbiology, Faculty of Biochemistry, Biophysics and Biotechnology, Jagiellonian University, 30-387 Kraków, Poland; Department of Biology, College of Science, Salahaddin University-Erbil, 44002 Erbil, Kurdistan Region, Iraq; Department of Community Health, College of Health Technology, Cihan University-Erbil, 44001 Erbil, Kurdistan Region, Iraq; Department of Analytical Chemistry and Biochemistry, Faculty of Materials Science and Ceramics, AGH University of Science and Technology, 30-059 Kraków, Poland; Laboratory of Protein Structure, International Institute of Molecular and Cell Biology, 02-109 Warsaw, Poland; Protein Crystallography Research Group, Malopolska Centre of Biotechnology, Jagiellonian University, 30-387 Kraków, Poland; Department of Biochemistry and Molecular Genetics, University of Virginia School of Medicine, Charlottesville, VA 22903, USA; Virogenetics Laboratory of Virology, Malopolska Centre of Biotechnology, Jagiellonian University, 30-387 Kraków, Poland; Department of Microbiology, Faculty of Biochemistry, Biophysics and Biotechnology, Jagiellonian University, 30-387 Kraków, Poland; Laboratory for RNA Biology, Berlin Institute for Medical System Biology, Max Delbrück Center for Molecular Medicine in the Helmholtz Association, 10115 Berlin, Germany; Department of Analytical Biochemistry, Faculty of Biochemistry, Biophysics and Biotechnology, Jagiellonian University, 30-387 Kraków, Poland; Laboratory for RNA Biology, Berlin Institute for Medical System Biology, Max Delbrück Center for Molecular Medicine in the Helmholtz Association, 10115 Berlin, Germany; Laboratory of Protein Structure, International Institute of Molecular and Cell Biology, 02-109 Warsaw, Poland; Protein Crystallography Research Group, Malopolska Centre of Biotechnology, Jagiellonian University, 30-387 Kraków, Poland; Helmholtz Zentrum München, 85764 Neuherberg, Germany; Bavarian NMR Center, Department of Chemistry, Technical University of Munich, 85748 Garching, Germany; Department of Analytical Chemistry and Biochemistry, Faculty of Materials Science and Ceramics, AGH University of Science and Technology, 30-059 Kraków, Poland; Helmholtz Zentrum München, 85764 Neuherberg, Germany; Bavarian NMR Center, Department of Chemistry, Technical University of Munich, 85748 Garching, Germany; Virogenetics Laboratory of Virology, Malopolska Centre of Biotechnology, Jagiellonian University, 30-387 Kraków, Poland; Virogenetics Laboratory of Virology, Malopolska Centre of Biotechnology, Jagiellonian University, 30-387 Kraków, Poland

## Abstract

Coronaviruses modify their single-stranded RNA genome with a methylated cap during replication to mimic the eukaryotic mRNAs. The capping process is initiated by several nonstructural proteins (nsp) encoded in the viral genome. The methylation is performed by two methyltransferases, nsp14 and nsp16, while nsp10 acts as a co-factor to both. Additionally, nsp14 carries an exonuclease domain which operates in the proofreading system during RNA replication of the viral genome. Both nsp14 and nsp16 were reported to independently bind nsp10, but the available structural information suggests that the concomitant interaction between these three proteins would be impossible due to steric clashes. Here, we show that nsp14, nsp10, and nsp16 can form a heterotrimer complex upon significant allosteric change. This interaction is expected to encourage the formation of mature capped viral mRNA, modulating nsp14’s exonuclease activity, and protecting the viral RNA. Our findings show that nsp14 is amenable to allosteric regulation and may serve as a novel target for therapeutic approaches.

## Graphical Abstract

**Graphical Abstract:**
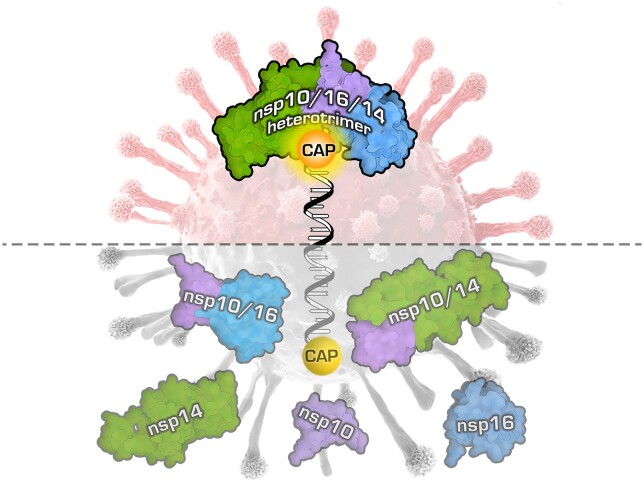

## Introduction

Upon release into the cytoplasm, the coronaviral genomic RNA (gRNA) serves directly as a substrate to the translational machinery. The first and only product of gRNA translation is a large, nonfunctional 1a/1ab polyprotein. To acquire functionality, it undergoes maturation through autoproteolytic cleavage mediated by two viral proteases, namely the main protease (M^pro^) and papain-like protease (PL^pro^). This leads to the generation of a set of nonstructural proteins (nsp) which are responsible for the viral replication process and remodeling of the intracellular environment. Once nsps reshape the cell to better serve the viral replication, gRNA is being replicated, and at the same time a set of subgenomic mRNAs is produced in a peculiar, discontinuous transcription process. These subgenomic mRNAs are monocistronic and serve as templates to produce structural and accessory proteins required for the formation, assembly, and release of progeny viruses (1). While the functions of individual nsps have been previously characterized, the coordinated action of these multifunctional components remains incompletely understood and requires further investigation. Works by Gao *et al.* (2), Yan *et al.* (3), Wang *et al.* (4) and Kabinger *et al.* (5) shed light on the scaffold of the replication complex formed by the nsp12 polymerase and two co-factors (nsp7 and nsp8), which creates the functional machinery with the ability to replicate viral RNA. Next, an extended elongation complex was described, where nsp12/7/8 is accompanied by the nsp13 helicase. This complex is suggested to serve as the basic replication module (6).

Coronaviruses are known for their large genomes, requiring high-fidelity replication to maintain their integrity; although the SARS-CoV-2 nsp12 polymerase exhibits high processivity, it is prone to errors and lacks the required fidelity (7). Consequently, coronaviruses have evolved an intrinsic proofreading system (8–10), which involves several proteins, with particular emphasis on nsp14, which harbors an N-terminal 3′-5′ exonuclease domain responsible for excising inaccurately incorporated nucleotides from the 3′ terminus of newly synthesized RNA. The enzyme has been also proposed to play a role during the discontinuous replication of coronaviruses. Nsp14 associates with the replicatory complex, with nsp10 as a co-factor enabling its exoribonuclease activity (8–10).

Nsp14 is a multifunctional protein containing an N-terminal exonuclease domain (ExoN) and C-terminal guanine-N7-methyltransferase (N7-MTase) domain. Together with nsp16, this domain performs double methylation of the RNA cap, a process vital for the function and integrity of viral mRNAs (8). The methylation allows for translation initiation and protects viral mRNA from recognition as foreign by innate immunity (11). Cap formation is a tightly regulated process consisting of four consecutive enzymatic reactions. First, the kinase-like nidovirus RdRp-associated nucleotidyltransferase (NiRAN) domain (12) of nsp12 transfers the RNA to the amino terminus of nsp9, forming a covalent RNA–protein intermediate. Next, the NiRAN domain transfers the RNA to GDP, forming the core cap structure GpppA-RNA (13). Subsequently, the GpppA is methylated at the N7 position by the nsp14 N7-methyltransferase domain (^7Me^GpppA), and the ribose of the first ribonucleotide is methylated at the 2′-O-position by the nsp16 2′-*O*-methyltransferase (14–16). During capping, the activity of the exonuclease domain of nsp14 must be modulated to prevent excessive destruction of the RNA. The described process results in a functional cap (^7Me^GpppA_2′OMe_), completing the genome replication. While there is a good structural understanding of the interactions between nsp12, nsp13, and nsp14 during replication (6,17), for nsp16 the methylation process is not fully understood. The spatial proximity of nsp14 and nsp16 should improve efficiency of the process. However, the available data contests such an interaction, as previous computational analyses utilizing available structural information on nsp10/14 and nsp10/16 complexes suggested that concurrent binding of both proteins to nsp10 might be unfeasible due to steric clashes (18).

In this study, we investigated the interaction of the two proteins (nsp14 and nsp16) bridged with their co-factor nsp10. Through the application of orthogonal methodologies, we have successfully captured the simultaneous presence and interactions of all three proteins and comprehensively studied the modulations of their enzymatic activities upon the complex formation.

An in-depth characterization of this co-dependency is warranted to define the role of this methylation complex in the SARS-CoV-2 virus’ replication machinery. Interestingly, both nsp14 and nsp16 interact with nsp10 via canonical protein-protein interfaces, characterized by deep-seated lipophilic residues and solvent-shielded hydrogen bonds. The element triggering the steric interference between nsp14 and nsp16, when considering their attachment to a single nsp10 molecule, is an unusual N-terminal lid domain within nsp14. This lid is mostly devoid of secondary structure and lacks the protein-protein interaction complementarity. Notably, recent structures of nsp14 without nsp10 show substantial conformational rearrangements of the lid region, consistent with its structural flexibility (19–21). These findings led us to hypothesize that a localized structural alteration in the N-terminal lid region of nsp14 might be possible, and we show the formation of a heterotrimeric nsp10/16/14 complex.

Further, we analyzed the available data on the formation of the replication complex, with a particular focus on the flexible and disordered structure of the nsp14 lid (19–21). Building upon our data, which suggests the possibility of nsp14 and nsp16 co-interacting with nsp10, we formulated a hypothesis proposing that, thanks to a rearrangement of the N-terminal lid of nsp14 to expose the nsp10–nsp16 interface, these three proteins form a stable complex to facilitate replication and enhance the RNA cap methylation of the coronaviral genome. To further explore this hypothesis, we established an *in vitro* model to reconstitute the potential protein complex, successfully demonstrating its assembly and structural organization. Remarkably, while the methyltransferase activity of nsp14 and nsp16 in the complex remained relatively unchanged, we observed a modulation of nsp14’s exonuclease activity upon the complex formation, what may be essential for the regulation of the functional activity of nsp14 in the cellular microenvironment.

Our findings contribute to the current understanding of the intricate interplay among nsp14, nsp10 and nsp16 during the replication process, particularly in the context of RNA cap methylation in the coronaviral genome. This research may have implications for the deeper understanding of the coronavirus’ biology and development of novel targeted therapeutic strategies for the control of coronaviral infections.

## Materials and methods

### Protein expression and complex purification

Constructs of nsp10, comprising amino acids 4254–4392; nsp14wt, comprising amino acids 5926–6452; and nsp16, comprising amino acids 6799–7096 of SARS-CoV-2 polyprotein 1ab optimized for expression in *E. coli* were ordered from GeneArt and individually subcloned into expression vector pETDuet-1, where fused TEV cleavage site (ENLYFQ/G) is added after His-tag for each of the proteins. In order to evaluate the nsp14’s influence on the heterotrimer formation, we have generated three additional variants of nsp14: mutated exonuclease active sites D90A and E92A (nsp14cat) (22); lacking the methyltransferase domain and keeping the exonuclease activity (nsp14ExoN); and deprived of the N-terminal 50 amino acids forming the lid (nsp14Δ) (Supplementary Table S1). Heterotrimers and heterodimers were obtained from co-expressions of individual protein plasmids in *E. coli*. All reported complexes were expressed and purified according to the protocol below. Nsp14cat was purified according to the protocol described by us (20).

Transformed BL21 (DE3) *E. coli* cells were grown in Lennox Broth (LB) medium (Bioshop) supplemented with 100 μg/ml of ampicillin (Sigma) at 37 °C overnight and used as a starter culture for large-scale expression in Terrific Broth (TB) medium (Bioshop). After the culture reached OD_600_ = 1.2–1.4, cultures were cooled down and induced with 0.5 mM isopropyl-d-1-thiogalactopyranoside (IPTG; Sigma) for protein expression at 18°C for 16 h. Bacterial pellets were collected by centrifugation at 6000 rpm for 10 minutes at 4 °C, resuspended in lysis buffer (50 mM Tris–HCl pH 8.5, 300 mM NaCl, 5 mM MgCl_2_, 10 mM imidazole, 0.01% Triton X-100, 5% v/v glycerol, 5 mM β-mercaptoethanol (β-ME), 10 ug/ml DNAse I (Roche) and protease inhibitor cocktail (Thermo Fisher Scientific) and disrupted by sonication at 80% amplitude with 3 s ON and 3 s OFF pulse cycles, for 15 min at 10°C. The lysed sample was clarified by centrifuging for 1 h at 25 000 rpm at 4°C. The supernatant was collected and incubated for 2 h at 4°C with 2 ml of Ni-NTA Agarose resin (Jena Bioscience), pre-equilibrated with the lysis buffer. Purification was carried out in a gravity-flow column. Resin was washed with 50 bed volume (BV) of buffer A (50 mM Tris–HCl pH 8.5, 300 mM NaCl, 5 mM MgCl_2_, 5 mM β-ME, 10 mM imidazole) and 20 BV of buffer B (50 mM Tris–HCl pH 8.5, 300 mM NaCl, 5 mM MgCl_2_, 5 mM β-ME, 20 mM imidazole). The protein complex was eluted with buffers C, D and E (50 mM Tris–HCl pH 8.5, 150 mM NaCl, 5 mM MgCl_2_, 5 mM β-ME, 100 mM (C)/250 mM (D)/350 mM imidazole (E), 5 × 2 BV each). The eluted fractions were concentrated to 5 ml, loaded and ran at a flow rate of 200 μl/min on a HiLoad 26/600 Superdex 200 prep grade column (GE Healthcare) equilibrated with the SEC buffer (50 mM Tris–HCl pH 8.5, 150 mM NaCl, 5 mM MgCl_2_, 5% v/v glycerol, 5 mM β-ME). Once confirmed by SDS-PAGE, the nsp10/16/14 protein heterotrimer complex fractions were collected. TEV protease and β-ME were added and incubated with gentle rocking at 4°C for approximately 12h to cleave the His-tags. When the His-tags have been cleaved from the heterotrimer complex, reverse binding was performed to clear the His-tags and other impurities by incubating for 15 min at 4°C with 500 μl of Ni-NTA Agarose (GE Healthcare), pre-equilibrated with the SEC buffer. The sample was separated with a gravity-flow column, and the Ni-NTA agarose was washed three times with the SEC buffer with 150 mM NaCl and finally with SEC buffer with 300 mM NaCl. The protein complex was collected, concentrated, loaded, and ran at a flow rate of 100 μl/min on a Superdex 200 Increase 10/300 GL column (GE Healthcare) equilibrated with the final SEC buffer (20 mM HEPES pH 7.5, 150 mM NaCl, 5 mM MgCl_2_, 1 mM tris(2-carboxyethyl)phosphine (TCEP)). The protein complex was collected and concentrated at 5 mg/ml. Aliquots were snap-frozen and stored at -80°C for further applications and measurements. Examples of the obtained purity of single proteins and respective protein complexes purified from their co-expression are shown by SDS-PAGE, in Supplementary Figure S1.

### Mass photometry

Mass photometry (MP) experiments were performed using OneMP instrument (Refeyn, Oxford, UK). First, Rectangular 24 × 50 mm coverslips were prepared by repetitive rinsing with water, ethanol, and isopropanol. Next, the cleaned coverslips were dried with clean nitrogen. To calibrate the measurements, molecular weight references diluted in the final protein buffers were measured first. MP signals for sufficiently diluted protein samples were recorded for 100 s. Raw MP data were processed in DiscoverMP software (Refeyn, Oxford, UK) and subsequently plotted as molar mass distribution histograms using PhotoMol server (23).

### Liquid chromatography tandem mass spectrometry (LC–MS/MS)

Protein identification from gel bands was performed at the Proteomics and Mass Spectrometry Core Facility, Malopolska Centre of Biotechnology, Jagiellonian University, Kraków, Poland. Samples were prepared, tested, and analyzed as described in Pabis *et al.* (24) with minor changes. Briefly, gel bands were destained by alternating washing with 25% and 50% acetonitrile (ACN) in 25 mM ammonium bicarbonate (ABC). Then, protein reduction was performed with 50 mM dithiothreitol (DTT) in 25 mM ABC (45 min of incubation at 37°C) followed by alkylation with 55 mM iodoacetamide with 1 h incubation at room temperature (RT) in the dark. In the next steps, gel bands were washed with 50% ACN in 25 mM ABC, dehydrated in 100% ACN, dried and rehydrated in 20 μl of trypsin solution (10 ng/μl in 25 mM ABC). After rehydration, 40 μl of 25 mM ABC was added and samples were left for overnight incubation at 37°C. Protein digestion was stopped by adding trifluoroacetic acid (TFA) at a concentration of about 0.5%. Peptides in the solution were collected and additionally extracted from the gel by dehydration with 100% ACN. The obtained peptide mixtures were dried and suspended in a loading buffer (2% ACN with 0.05% TFA) for LC–MS/MS analysis, carried out with a nanoHPLC (UltiMate 3000 RSLCnano System, Thermo Fisher Scientific) coupled to a Q Exactive mass spectrometer (Thermo Fisher Scientific). Peptides were loaded onto a trap column (Acclaim PepMap 100 C18, 75 μm × 20 mm, 3 μm particle, 100 Å pore size, Thermo Fisher Scientific) at a flow rate of 5 μl/min and separated on an analytical column (Acclaim PepMap RSLC C18, 75 μm × 500 mm, 2 μm particle, 100 Å pore size, Thermo Fisher Scientific) at 50°C with a 60 min gradient of ACN, from 2% to 40%, in the presence of 0.05% formic acid at a flow rate of 250 nl/min. The eluting peptides were ionized in a Digital PicoView 550 nanospray source (New Objective) and measured using Q Exactive operated in a data-dependent mode. A Top8 method was used with 35 s of dynamic exclusion. MS and MS/MS spectra were acquired at resolutions of 70 000 and 35 000, respectively. The ion accumulation times were adjusted to ensure parallel filling and detection. The acquired LC–MS/MS data were processed with the use of Proteome Discoverer platform (v.1.4; Thermo Scientific) and searched with an in-house MASCOT server (v.2.5.1; Matrix Science, London, UK) against the database of common protein contaminants (cRAP database, https://www.thegpm.org/crap/) with manually added sequences for the proteins of interest. The following parameters were applied for the database search: enzyme: trypsin; missed cleavages: up to 1; fixed modifications: carbamidomethyl (C); variable modifications: oxidation (M); peptide mass tolerance: 10 ppm; fragment mass tolerance: 20 mDa. Additionally, the SwissProt database, restricted to *E. coli* taxonomy, was searched to assess contamination with host proteins.

### Stoichiometry determination

For protein quantitation, sample separation was carried out following a simple protocol using the Prominence HPLC system (2 × LC-20AD pumps, SPD-M20A diode array detector, DGU-20 degasser, all from Shimadzu Corp., Kyoto, Japan). For the gradient separation, a Kinetex 2.6 μm/100A C18 100 mm/2.1 mm ID HPLC column was used (00D-4462AN, Phenomenex, Torrance, CA, USA). Solvents used for separation: A = water + formic acid (99.9:0.1, v/v), B = ACN + formic acid (99.9:0.1, v/v). All solvents were supplied by a Merck local distributor (Merck KgaA, Darmstadt, Germany). The gradient was set as follows: t (time) = 0 min, 25% B; *t* = 20 min, 75% B; *t* = 20.5 min, 90% B; *t* = 25 min, 90% B; *t* = 25.5 min, 25% B; *t* = 35 min, 25% B (end). The flow rate was set to 0.3 ml/min. Diode array detector settings: λ = 200–350 nm (deuterium lamp only), sampling frequency: 5 Hz. Data acquisition and data processing were controlled by LCsolution software, version 1.25 (Shimadzu Corp., Kyoto, Japan). Resulting data from λ = 280 nm chromatogram were corrected by the molar extinction coefficients calculated for each protein (using ProtParam tool available at web.expasy.org/protparam/web page). Peak symmetry and other visible parameters were also verified using chromatograms acquired at other λ.

To confirm protein content under every chromatographic peak taken for protein quantitation, mass spectrometry-based identification of each fraction composition was used. The protocol for protein identification, applied with minor changes, is available elsewhere (25). Briefly, fractions acquired during protein separation were freeze-dried using a CentriVap system (Labconco, Kansas City, MO, USA) and re-dissolved in 70 μl 50 mM ABC (pH 7.8). Next, reduction and alkylation of cysteine residues were done using DTT and following iodoacetamide 50 mM ABC solutions (both reagents: 5 mM final concentrations). In both cases, 10 min incubation at 80°C with shaking was applied. After cooling down, trypsin (Gold-MS grade, Promega, Madison, WI, USA) was added at a final concentration of 2 pmol per sample. Samples were incubated overnight at 37°C with shaking, then freeze-dried again and re-dissolved in 30 μl of 4% ACN/water solution acidified by 0.1% formic acid (v/v/v). NanoLC–MS/MS analyses were performed on an Ultimate 3000 system (Thermo, Waltham, MA, USA) connected on-line to AmaZon SL, equipped with a nanoFlowESI ion source (Bruker-Daltonics, Bremen, Germany). Parameters of nanoLC system: column Acclaim PepMap100, C18, λ = 10 cm/75 μm I.D., precolumn PepMap100, C18, λ = 1 cm/1 mm I.D., gradient settings: solvent A = water with 0.1% formic acid (v/v), solvent B = ACN with 0.1% formic acid (purity: MS-grade; Merck KGaA, Darmstadt, Germany), *t* = 0 min, 6% B; *t* = 5 min, 6% B; *t* = 50 min, 55% B; *t* = 50.1 min, 80% B; *t* = 53 min, 80% B; *t* = 54 min, 6% B; *t* = 58 min, 6% B (end). Sample injection volume was usually in the range 3–5 μl. Flow rate: 300 nl/min, flow rate for sample introduction on precolumn: 30 μl/min. Mass spectrometer settings were as follows: capillary voltage: 4200 V; heated capillary temperature: 150°C; MS scan range: 375–1600 *m*/*z*; MS/MS scan range: 200–2000 *m*/*z*; resolution: enhanced; scanning frequency: ca. 1 Hz; ICC (Ion Charge Control): 250 000 ions/trap cycle; fragmentation ions selection range: 450–1600 *m*/*z* (with minor exclusions); minimal ion intensity selected for fragmentation: at least 1 × 10^6^ units. Both instruments were run under the HyStar version 4.1 SR1 (Bruker-Daltonics, Bremen, Germany). Data analysis was performed in Bruker's Compass DataAnalysis 4.4 SR1. Acquired data were converted into mgf files using built-in scripts, introduced into Mascot search engine (version 2.6, Matrixscience, London, UK), and searched against SwissProt and an in-house created database. Mascot settings: enzyme: trypsin; missing cleavages: 1; taxonomy: all; fixed modifications: carbamidomethylation; variable modifications: oxidation-Met; peptide tolerance: 1.2 Da; #13C: 1; MS/MS tolerance: 0.6 Da; peptide charge: +1, +2, +3; instrument: ion-trap.

### MicroScale thermophoresis (MST)

His-tag proteins were labeled with the Monolith His-Tag Labeling Kit RED-tris-NTA 2^nd^ Generation (Nanotemper, MO-L018) according to the manufacturer's guidelines. Labeled proteins were diluted in PBS containing 0.05% Tween-20 up to 80 nM concentration and mixed with increasing concentrations of tested ligands. Samples were allowed 30 min incubation at RT before measurement. Measurements were performed on Monolith NT.115 in independent duplicate dilution series using 80% Excitation Power and high MST Power in Monolith NT.115 Capillaries (MO-K022). Data were then fitted using MO.Affinity Analysis software *K*_D_ fit.

### Nanoscale differential scanning fluorimetry (nanoDSF)

NanoDSF was performed in standard capillaries using Tycho NT.6 equipment. Proteins and their respective complexes were measured at 1 mg/ml using default ramp temperature in dedicated capillaries after 15 min at RT incubation. The resulting melting temperatures were reported as the first derivative of the fluorescence ratio.

### Methyltransferase activity

The methyltransferase activity of nsp10/16/14wt, nsp10/16/14cat, and nsp10/16/14ExoN heterotrimers were measured using the EPIgeneous Methyltransferase kit from Cisbio (62SAHPEB) as previously described (26). Individual kit reagents were reconstituted according to the manufacturer's instructions. Briefly, the methyltransferase reaction was incubated for 20 min at RT in 8 μl reaction volume with 100 nM nsp10/16/14wt or mutated heterotrimer, 1 μM Ultrapure SAM (from the Cisbio's kit), 187.5 μM RNA cap analog (GpppA or GpppG, New England Biolabs S1406S and S1407S, respectively) or 18.75 μM Cap 0 RNA oligo (TriLink, produced on request) in reaction buffer consisting of 20 mM Tris–HCl pH 7.4, 150 mM NaCl and 0.5 mM DTT. The reaction was quenched by adding 2 μl of 5M NaCl followed by the addition of 2 μl Detection Buffer 1 (from the Cisbio's kit) to the reaction mixture. After 10 min, 4 μl of 16× SAH-d2 conjugate solution (from the Cisbio's kit) was added. After 5 min, 4 μl of 1× a-SAH Tb Cryptate antibody solution (from the CisBio's kit) was added to the reaction mixture and incubated for 1 hour at RT. HTRF measurements were performed on a SpectraMax iD5 plate reader (Molecular Devices) according to the manufacturer's guidelines (excitation λ 340 nm, emission λ 665 and 620 nm, top mode, 100 flashes, optimal gain, z position calculated from the negative control (no enzyme), lag time of 60 μs and the integration time of 500 μs). The resulting data were background-subtracted and normalized as follows: a ratio of signal at λ 665 nm and 620 nm was calculated; the data were background-corrected on the averaged signal for the buffer control; the data was normalized for each series individually on the wells not containing the enzyme. Sinefungin (Sigma, S8559) was used as a positive control.

### Nsp14 non-methylated oligo synthesis

The substrate for SARS-CoV-2 nsp14 was synthesized as described previously (26). Briefly, DNA oligos were incubated in a buffer containing 10 mM Tris–HCl, pH 7.5, 50 mM NaCl, and 1 mM EDTA at 95°C for 2 min, and then cooled down to 20°C, at 1°C/min. Transcription and RNA capping were conducted with the HiScribe T7 High Yield RNA Synthesis kit (NEB, E2040S). The reaction mixture contained all NTPs at 10 mM except GTP, which was added at 0.75 mM and GpppA cap analog at 9.25 mM. The reaction was conducted for 16 h at 37°C and the RNA was purified with a Monarch RNA cleanup kit (NEB, T2040S).

### Binding and exonuclease activity assays

Fluorescently labeled RNA oligonucleotides (CoV-RNA1-A 5′ FAM-AAAAAAAAAAACGCGUAGUUUUCUACGCG 3′ and CoV-RNA1-G 5′ FAM-GGGGGGGGGGGCGCGUAGUUUUCUACGCG 3′) for exonuclease assays were synthesized by Future Synthesis. RNA oligonucleotides were dissolved in DEPC-treated water. Exonuclease assays were performed with a protein concentration of 1.6 μM and RNA substrate concentration of 1.6 μM in a reaction that contained 20 mM HEPES (pH 7.5), 100 mM NaCl, 5% glycerol, 10 mM MgCl_2_, 5 mM β-ME. The reactions were initiated by the addition of protein at 37°C and stopped at selected time points by the addition of an equal volume of 100% formamide that contained Orange G dye and boiling the samples for 5 min at 95°C. The samples were analyzed on 20% TBE-Urea polyacrylamide gel at a constant power of 18 W for 45 min. The products were visualized with the Amersham Typhoon RGB Biomolecular Imager with Image Quant Total Lab software (GE Healthcare).

### Nuclear magnetic resonance (NMR)

^2^H^15^N-nsp10 was expressed in M9 minimal media supplemented with ^15^N-NH_4_Cl_2_ in 100% D_2_O. The purification was done as reported previously (7). Purified ^2^H^15^N-nsp10 was used at 100 μM for NMR experiments. NMR spectra were collected on Bruker 800 and 1200 MHz NMR spectrometers equipped with a triple resonance cryoprobe. Non-labeled nsp16 and nsp14cat were titrated and added to the ^2^H^15^N-nsp10 with a 2-fold molar excess. Despite molar excess it was not possible to completely saturate nsp10 due to aggregation and solubility limit of the nsp16. Interpretation of spectra is based on, clearly distinguishable, bound fraction of nsp10. The interaction between nsp10 and nsp14 was not observed in the NMR spectra. This surprising fact can be explained by weaker nsp10-nsp14 affinity, and may be affected by NMR conditions (concentrations, temperature, buffer components) or alternate folding of the N-terminal lid of nsp14 that refolds to bind nsp10 only very slowly or in a presence of nsp16. This second possibility is corroborated by our recent structure of apo-nsp14 where the lid completely refolds to accommodate the nsp10 (20).

### Molecular dynamics simulations (MD)

We simulated three systems: nsp14, nsp10/14 complex, and the nsp10/16/14 complex. The nsp14 protein was taken from the nsp10/14 complex (PDB ID: 7DIY). In the case of the heterotrimer, we generated the complex by aligning the nsp10/14 complex (PDB ID: 7DIY) with a nsp10/16 complex (PDB ID: 6W4H). To avoid steric clashes, the lid region had to be displaced by rotating along the Tyr51 main chain. No other alterations were made to the structures. The MD simulations were performed with the AMBER 20 (27) software suite, and the proteins were parameterized using the ff14SB (28) force field. The systems were solvated in explicit water (TIP3P (29)) using an octahedron water box with a minimum distance of solute and box edge of 15 Å. The systems were neutralized using Na^+^ and Cl^−^ ions. The systems were minimized (1′000 steps of steepest descent) and heated to 300 K in several steps using a *langevin* thermostat. After equilibration (4 ns including heating) the production runs were performed. For nsp10/14 and nsp14, we performed 2 μs of simulation. In the case of heterotrimer complex we conducted three independent simulations (from different starting conditions) summing to 1.7 μs. The trajectories were analyzed using *pytraj*. For the RMSF analysis a per atom RMSF was calculated for all cases and the mean for each residue was taken. The hydrogen bonds were calculated based on a distance criterion of 3 Å and an angle cutoff of 135°.

### Small-angle X-ray scattering (SAXS)

Samples were measured in SEC-SAXS mode at BM29/ESRF, Grenoble, France (session ID: MX2341). Samples (100 μl) were measured on Agilent AdvanceBio SEC 300 with 50 mM Tris–HCl pH 8.5, 150 mM NaCl, 5 mM MgCl_2_ and 2 mM β-ME running phase at 0.16 ml/min flow rate. The measurements were performed at 0.99 Å wavelength. The sample-to-detector distance was set at 2.83 m with a Pilatus2M detector for data acquisition. Further data analysis was performed using the ATSAS 3.1.3 software package. Rg was measured using Primus using a qRg range not higher than 1.5. The real-space parameter determination was assessed using GNOM software. The molecular weights were assessed using the Bayesian Inference mode of Primus considering Qp, MoW, Vc, size and shape values. Molecular envelopes were generated using DAMMIF 10 rounds finished with one DAMMIN run. They were visualized using SASpy. High-resolution PDB structures were superimposed in their corresponding molecular envelopes using SASpy mode supalm. The model of nsp10/16/14wt heterotrimer was built using SRELFEX software using high-resolution structures of nsp10/16 (PDB ID: 6W4H) and nsp14 from PDB ID: 5C8U.

### Negative-staining transmission electron microscopy (NS-TEM)

NS-TEM measurements were done in Formvar/carbon-supported 400 mesh copper grids suspended in air with a negative lock tweezer. The purified protein complex (0.03 mg/ml) was applied on glow-discharged Formvar/carbon-supported 400 mesh copper grids and negatively stained with 1% neutralized uranyl-acetate. Grids were imaged using a JEOL JEM 2100HT electron microscope (Jeol Ltd, Tokyo, Japan) at an accelerating voltage of 200 kV. Images were taken with a 4k × 4k camera (TVIPS) equipped with EMMENU software version 4.0.9.87.

### Cryo-electron microscopy cryo-EM) and subsequent analysis

Sample was applied on a 3 mm TEM grid (Quantifoil, Cu 2/1, mesh 200) and plunge-frozen in liquid ethane with Vitrobot mk IV (Thermo Fisher Scientific). Freezing parameters applied were: blot time of 2 s, single blot from both sides, 15 s incubation time, humidity 95%, and temperature 4°C. Prepared grids were clipped and transferred to a Titan Krios G3i microscope equipped with a K3 direct electron detector embed in a BioQuantum imaging filter setup (Gatan). A dataset of 8148 movies was collected with an average dose rate of 15.83 e^−^/px/s measured over vacuum. Each movie comprised 40 fractions with a total dose of 40.47 e^−^/Å^2^ again measured over vacuum. The defocus set ranged from –2.1 to –0.9 μm with the step of 0.3 μm. Movies were acquired in energy-filtered mode with a slit of 20 eV and pixel size of 0.86 Å. The acquisition was conducted with the use of EPU software (version 2.10.0.1941REL).

The collected dataset was processed in the cryoSPARC platform (versions from 3.2 to 4.0.0). After patch motion correction and patch CTF estimation, blob picking from 300 random micrographs was performed using particle diameters ranging from 80 to 160 Å. Picking resulted in a set of 168 819 particles, which were extracted with a box of 300 px, binned to 150 px, and 2D classified for further use in creating templates. Template picking from the whole dataset with 120 Å particle diameter resulted in a set of 2 534 664 particles. After extraction, particles were curated and subsequent 2D classified. Picked subset of particles was passed to train neural network picker. Obtained model resulted in 211 612 picked particles, extracted, 2D classified and passed to *ab initio* and homo refinement on 105 784 particles. The final density has resolution of 13.52 Å (at 0.143 FSC).

### Image processing and 3D reconstitution

Collected micrographs were processed using cryoSPARC 3.1.1. Initially, 9350 particles were picked from micrographs using Blob Picker. Picked particles were subjected to a template-free 2D classification, from which 1216 particles were selected and subjected to 3D reconstitution using the *ab initio* reconstitution job. The nsp10/16/14wt complex map derived from SAXS data was used for a rigid-body fit to a 3D-reconstruction map using Dock in map.

### Surface plasmon resonance (SPR)

The assay was performed on a Biacore S200 (GE Healthcare). Buffer exchange for both ligand and analyte were performed by diafiltration in Amicon Ultra 0.5 ml devices (Merck) to immobilization and assay buffer, respectively. Nsp14cat was immobilized on HC200M sensor (Xantec) in immobilization buffer (20 mM HEPES pH 7.3) by NHS-EDC crosslinking to ∼170 response units (RU). Assays were run in a multi-cycle scheme in assay buffer (10 mM HEPES pH 7.5, 150 mM NaCl, 5 mM MgCl_2_, 5 mM β-ME, 0.5 mg/ml BSA) at 30 μl/min flow rate. Each cycle consisted of a 120 s association step and 600 s dissociation step followed by surface regeneration (1-minute, 4 M MgCl_2_). Analyte (nsp10/16 complex) was injected at concentrations 0.5, 1, 2, 4, 8 and 16 μM. The affinity was calculated by fitting the binding level versus analyte concentration plot to the steady state affinity model.

## Results

### Exploring protein interactions: a computational perspective

**Figure 1.**
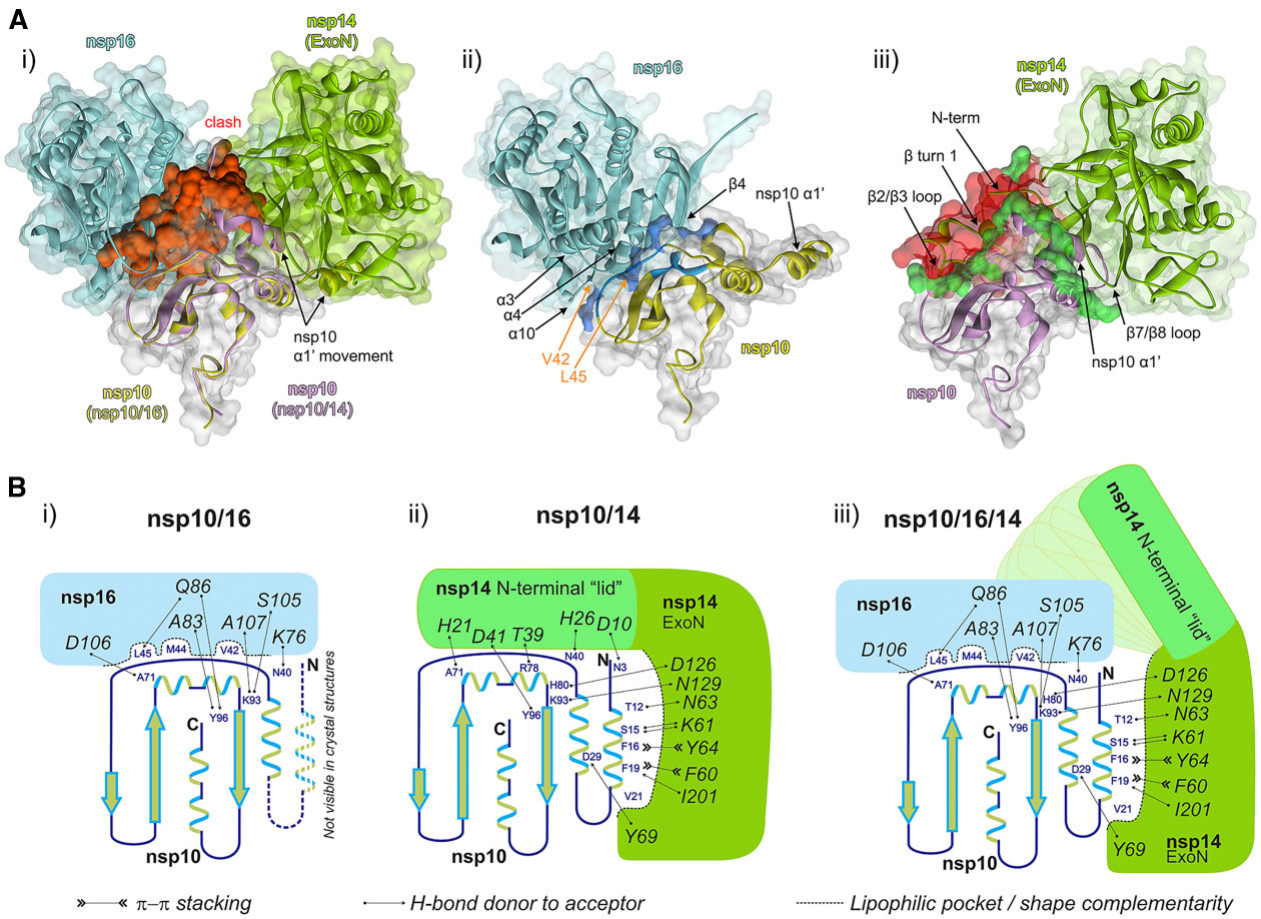
Nsp10/16/14 heterotrimer complex formation and the lid hypothesis. (**Ai**) Overlay of the available high-resolution structures of SARS-CoV-2 nsp10/14 and nsp10/16 complexes with nsp10-centered alignment of nsp10/16 (PDB ID: 6WVN) and nsp10/14 (PDB ID: 7DIY). Nsp16 is shown in cyan and the associated nsp10 is shown in yellow. Nsp14 exonuclease domain is shown in green and the associated nsp10 is in magenta. The structural clash between nsp14 and nsp10/16 surfaces is shown in red. (**Aii**) Interface between nsp10 and nsp16. The strong hydrophobic interaction between nsp10 and nsp16 are shown in blue. The nsp10 residues V42 and L45 are indicated by the orange arrows. (**Aiii**) The interface between nsp10 and nsp14. The sidechains participating in hydrogen bonds between nsp10 and nsp14 are shown in bright green. The N-terminal region of nsp14, where the β turn 1 and β2/β3 loop are located is where the steric clash (red) is localized. All panels: Variable orientations of nsp10 α1′-helices seen in respective complexes with nsp16 and nsp14 are shown. Arrows indicate major structural features constituting the interface. (**B**) Schematic representation of interactions guiding the affinities of nsp10, nsp14 and nsp16. (**Bi**) Nsp16 binds nsp10 at the site overlapping that involved in binding of the lid of nsp14, but nsp10/16 interaction is characterized by a well-developed interface involving deep lipophilic pockets and solvent-shielded hydrogen bonds. The α1 helix of nsp10 is not defined in the crystal structures of the nsp10/16 complexes (PDB IDs: 6W4H, 6YZ1), indicating it is flexible and not involved in binding. (**Bii**) The α1 helix of nsp10 provides several deeply buried lipophilic and π-stacking interactions with the exonuclease domain of nsp14, and the interaction may be described in terms of shape complementarity. In turn, the interaction of the N-terminal lid region (amino acids 1–50) of nsp14 with nsp10 shows poor shape complementarity and is characterized only by a low number of solvent-exposed hydrogen bonds. (**Biii**) The formation of the nsp10/16/14 heterotrimer complex is accompanied by the lid displacement and stabilization of the α1 helix.

Several high-resolution crystal structures are available for SARS-CoV-2 nsp10/14 and nsp10/16 complexes. Overlay of those by a common component (nsp10) demonstrates that a region comprising about 50 N-terminal residues of nsp14 overlaps with nsp16 at the surface of nsp10. The significant steric clash produced by this overlap would preclude the heterotrimer complex assembly mediated by the concomitant interaction of nsp14 and nsp16 with nsp10 (Figure 1Ai). Thus, to accommodate the formation of proposed heterotrimer, it would require a significant structural rearrangement within either nsp14 or nsp16.

Upon examination of the interactions between nsp10 and nsp16 (Figure 1Aii), and between nsp10 and nsp14 (Figure 1Aiii), it appears that the binding surface of nsp14 contains a weakly interacting N-terminal region, which we have previously shown to be flexible and disorganized (20). Based on the available binding interface data, there is a potential for the formation of a nsp10/16/14 heterotrimer, as nsp16 could potentially displace the N-terminal lid of nsp14 at the interface with nsp10, and this process might be energetically favorable. The interaction surface between nsp14 exonuclease domain (without the lid) and nsp10 would be sufficient to maintain the interaction and will not obstruct nsp16 binding (Figure 1B). This hypothesis is corroborated by the high structural diversity of the lid observed in our (PDB ID: 7R2V) and other X-ray structures (PDB IDs: 7DIY, 5C8U, 7N0B).

### Molecular dynamics simulation

**Figure 2.**
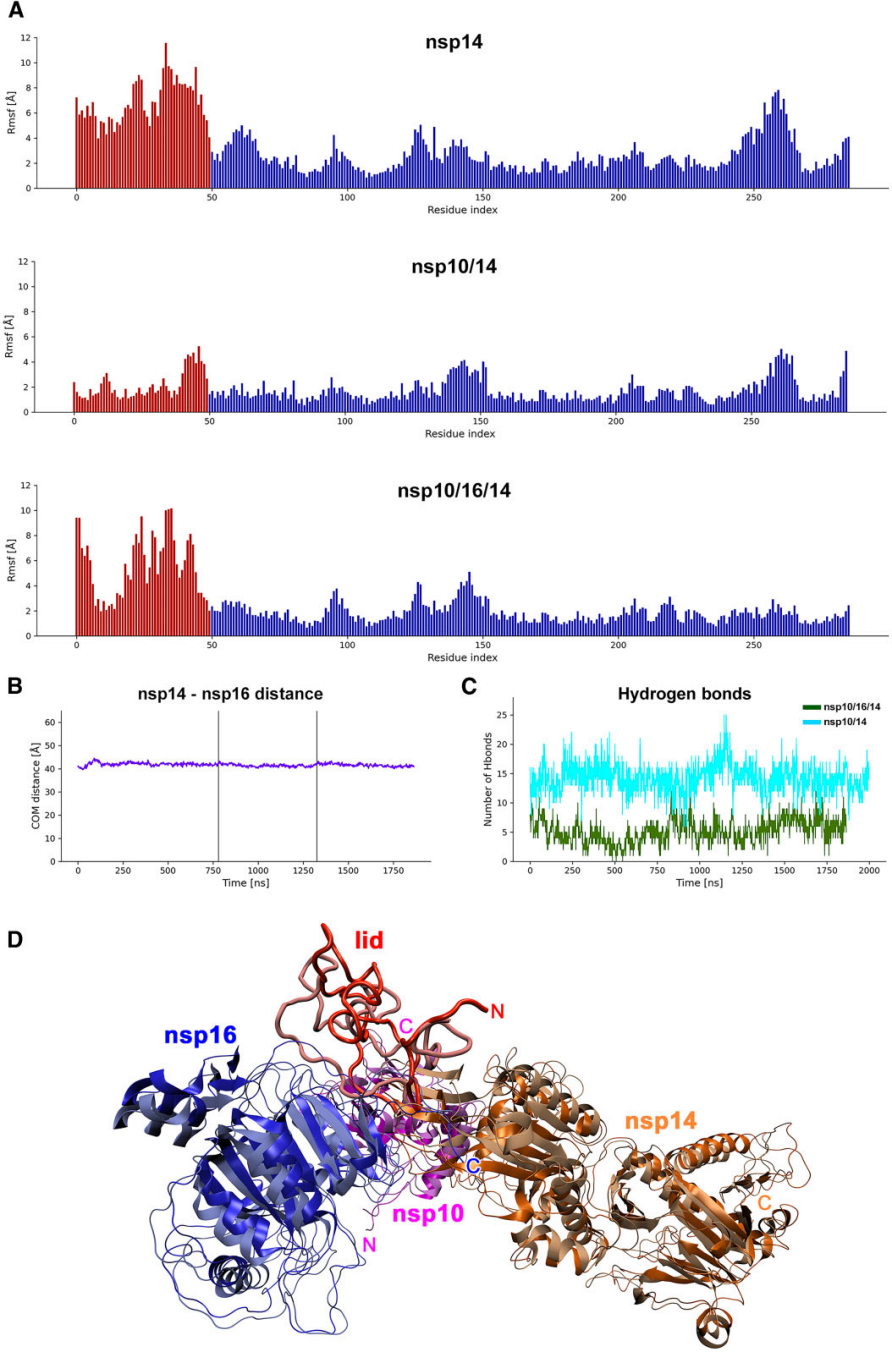
MD simulation. (**A**) RMSF against residue index for the nsp14 protein in each of the simulated cases with the lid part indicated as red bars. For each residue, the mean RMSF over all atoms is calculated. The lid part is highly flexible in free nsp14 simulation. Binding to nsp10 causes significant stabilization of this part. In the simulated heterotrimer, the lid is partially stabilized by interaction with nsp16 and additional interaction with the exonuclease domain but retains some degree of flexibility. (**B**) For the heterotrimer complex, the center of mass-distance between nsp14 and nsp16 stayed constant through the simulation time of total 1.7 μs and no dissociation happened. This indicates that nsp10 can accommodate both nsp14 (with lid readjustment) and nsp16 simultaneously. (**C**) The number of intermolecular hydrogen bonds between the lid part and the interacting proteins was calculated for nsp10/14 and the heterotrimer complex for each time frame. For nsp10/14, the lid was stabilized by on average 14 hydrogen bonds, and for the heterotrimer on average 5 hydrogen bonds appear between the proteins. (**D**) Heterotrimer structure simulated by MD. Nsp16 (blue), nsp10 (magenta), nsp14 (orange) and the lid region of nsp14 (red) are shown as ribbon plots. The first (dark hue, glossy) and last frame (light hue, matte) of the simulation are superimposed. The heterotrimer structure shows little structural deviations during the simulation except for the lid region that refolds visibly and a few loop regions. The overall shape of the complex remains stable.

In addition to a shared binding surface, the significant allosteric adaptation of nsp10 for exclusive binding of either nsp16 or nsp14 was suggested before (30). We noted that in both nsp10/14 and nsp10/16 structures, the regions forming intermolecular interfaces remain identical. To test if nsp10 would be capable of maintaining binding with both methyltransferases (assuming nsp14 lid refolding), we performed Molecular Dynamics (MD) simulations of the free nsp14, nsp10/14 and heterotrimer complex (Figure 2). The heterotrimer was modeled by superimposing nsp10 from nsp10/14 (PDB ID: 7DIY) and nsp10 from nsp10/16 (PDB ID: 6W4H) structures. The heterotrimer has nsp14 lid residues moved away from nsp10 (by the rotation of Tyr51 main chain) to allow for nsp16 binding. Our MD showed that the heterotrimer remained stable and did not dissociate in three independent MD runs amounting to 1.7 μs of simulation time. The lid region of nsp14 was quite flexible with no protein interactions and was stabilized in the nsp10/14 complex (see red bars in Figure 2A). In the heterotrimer, some flexible regions in the lid were also observable, but a new region stabilized by the interaction with nsp16 also appeared in this range. On average, the lid was stabilized by 5 intermolecular hydrogen bonds in the heterotrimer simulation. For nsp10/14 an average of 15 hydrogen bonds were found explaining the increased stability in this simulation.

Together, the MD simulations indicate that, upon lid rearrangement, the stable heterotrimer can exist and no allosteric adjustments of the nsp10 are necessary to bind both partners simultaneously. During the run, the rearranged lid formed a new interaction with nsp16 and the exonuclease domain of nsp14 that stabilized its structure. The degree of stabilization was smaller than observed for nsp10/14 complex. This could be due to the lid not being able to assume the global energy minimum within the duration of the simulation.

### In vitro heterotrimer formation

**Figure 3.**
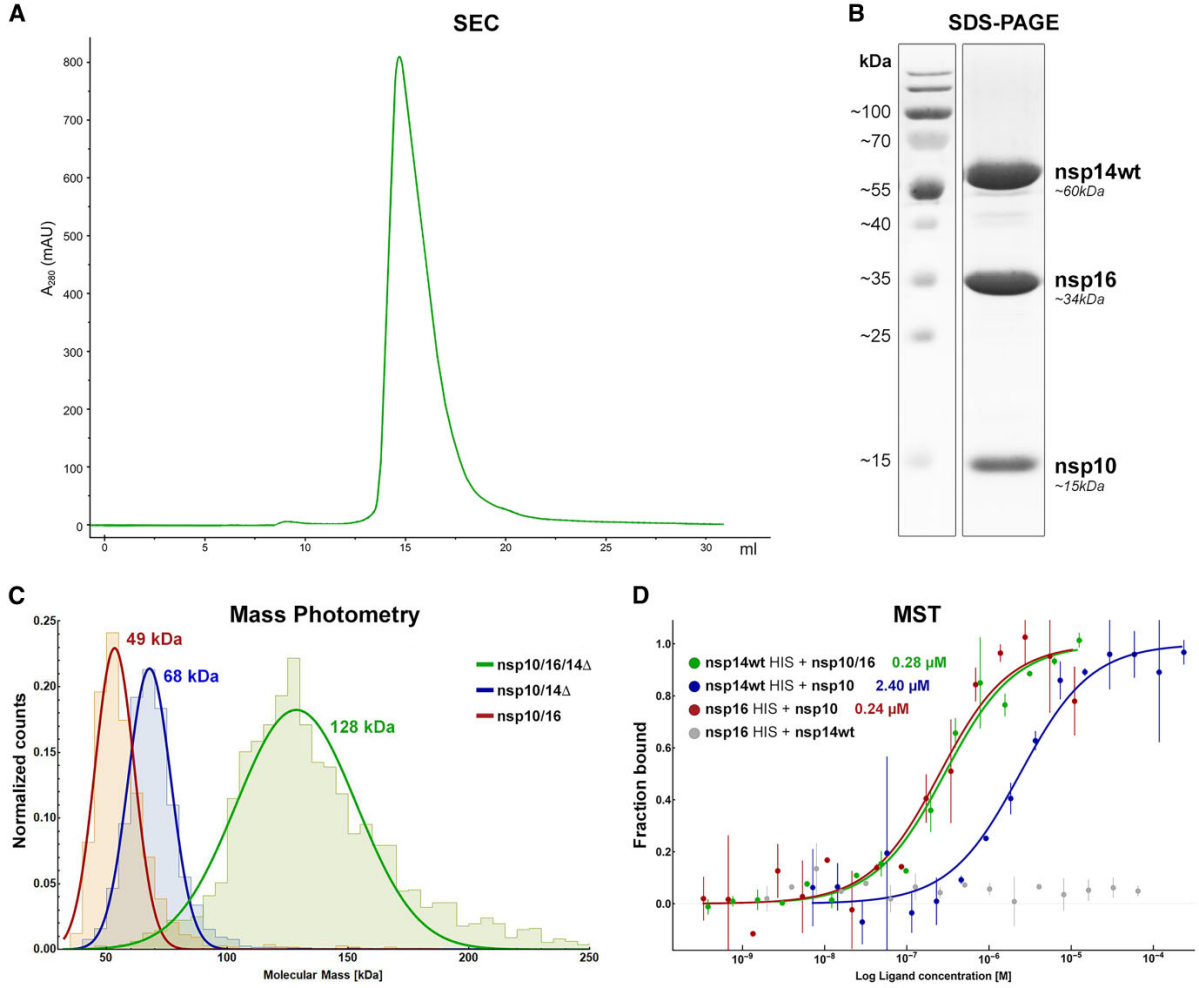
*In vitro* formation of the nsp10/16/14 heterotrimer. (**A**) SEC chromatogram for the heterotrimer complex (nsp10/16/14wt) eluting at 14.7 ml on a Superdex 200 Increase 10/300 column. (**B**) SDS-PAGE analysis of the main peak from the SEC run of the heterotrimer. Three major proteins corresponding in size to nsp10, nsp16, and nsp14 (MW of 15, 34 and 60 kDa, respectively) are present in roughly equal quantities. (**C**) MP determination of molecular masses for nsp10/16 (red - MW 49 kDa), nsp10/14Δ (blue - MW 68 kDa), and for the heterotrimer (green - MW 128 kDa). (**D**) MST analysis of the intra-complex affinities to form the heterotrimer nsp10/16/14wt (green), heterodimers nsp10/14wt (blue), and nsp10/16 (red). Nsp16 does not interact with nsp14wt (gray). Data are represented as mean (dots) with error bars. The binding model fit is represented as solid line.

The modeling and simulation data prompted us to experimentally verify the heterotrimer formation. To test the hypothesis and evaluate the interaction of nsp14 and nsp16 with nsp10, we have expressed four nsp14 variants: nsp14wt, full-length nsp14 protein; nsp14cat, nsp14 with mutated exonuclease active sites D90A and E92A; nsp14ExoN, nsp14 without the methyltransferase domain but retaining the exonuclease activity; and nsp14Δ, nsp14 lacking the N-terminal 50 amino acids (lid). First, we observed the formation of the nsp10/16/14wt heterotrimer by co-expressing the individual proteins into a single *E. coli* population, by Size Exclusion Chromatography (SEC, Figure 3A). The presence of nsp10, nsp16, and nsp14wt in the SEC peak fraction was confirmed by SDS-PAGE (Figure 3B). Next, to evaluate the nsp10/16/14wt heterotrimer formation by mixing the purified components, nsp14wt and nsp16 were separately expressed as His-tagged constructs and labeled with a high-affinity His-tag-specific fluorophore dye as the reporter for the MicroScale Thermophoresis (MST) experiments. Labeled nsp14wt was titrated with unlabeled nsp10, and a dose-dependent increase in the thermophoretic signal was observed, indicating an interaction with a *K*_D_ of 2.40 ± 0.20 μM (Figure 3D, blue). This is in agreement with the previously reported affinity with a *K*_D_ of 1.10 ± 0.90 μM (31). Every investigated variant of nsp14 was able to form nsp10/14 complexes. Nsp14Δ formed a stable complex with nsp10, with the *K*_D_ of 1.47 ± 0.42 μM, an increase in the affinity compared to the nsp10/14ExoN interaction with a *K*_D_ of 4.55 ± 2.68 μM (Supplementary Figure S2). The above data indicates that the N-terminal lid of nsp14 is largely dispensable for interaction with nsp10. The interaction is still possible and actually stronger for in nsp14Δ, where only the exonuclease domain provides binding surface. This is consistent with the possibility of lid rearrangement upon heterotrimer formation. The lack of secondary structures within the lid likely causes a significant entropic cost of the binding by restricting numerous degrees of freedom, thus offsetting the enthalpic contribution. Subsequently, we tested if nsp14Δ was still capable of interaction with nsp10/16 to form a heterotrimer. The complex was measured using Mass Photometry (MP) and MST. MP quantifies the so-called interferometric contrast (interference between the light scattered by the tested biomolecule and reflected by the surface used for measurements), which, unlike other techniques, is directly correlated to the molecular mass and not recalculated from, e.g. hydrodynamic radius (32). With MP, we were able to measure the approximate mass of the heterotrimer complex in solution, which is proportional to the recorded light scattering signal. Since we observed limited stability of nsp14wt in MP we used nsp14Δ for the experiments. Resulting histograms present sharp peaks for both nsp10/16 and nsp10/14Δ with fitted molecular weights of 49 and 68 kDa, respectively, and a distinct and broader, hence more dynamic, heterotrimer peak at 128 kDa (Figure 3C). This excludes the possibility of the heterotrimer being a mixture of co-migrating nsp10/14 and nsp10/16 heterodimers, we used MP to determine the molecular weights of the complexes directly in the solution.

To validate the specificity of our MST binding assay, we performed several control experiments. A well-defined nsp10-nsp16 interface yielded a *K*_D_ of 0.24 ± 0.01 μM (Figure 3D, red), whereas nsp14wt did not interact with nsp16 (Figure 3D, gray) in the absence of nsp10. When labeled nsp14wt was titrated with unlabeled nsp10/16 complex (Figure 3D, green), a dose-dependent increase in thermophoretic signal was observed. Fitting the experimental data allowed the determination of the *K*_D_ characterizing the interaction of nsp14wt with nsp10/16 at 0.28 ± 0.01 μM, a result consistent with the heterotrimer formation. The affinity of nsp14Δ for nsp10/16 was found to be 0.22 ± 0.03 μM, and was not significantly different from the affinity of nsp14wt for nsp10/16 at 0.28 ± 0.01 μM (Supplementary Figure S2A, green and Figure 3D, green), confirming that while the lid takes part in nsp10–nsp14 interactions, it does not significantly alter the heterotrimer formation.

To assess the stoichiometry of nsp10, nsp16 and nsp14wt in the complex, liquid chromatography-mass spectrometry (LC–MS) was employed. By analyzing the diode array detection signals, e.g. at 280 nm, with further MS-based identification of the proteins under each UV chromatographic peak, the stoichiometry was consistently established at 1.2:1:1 (nsp10:nsp16:nsp14wt) (Supplementary Figure S3 and Supplementary Table S2).

The binding of immobilized nsp14wt to nsp10/16 was further documented using Surface Plasmon Resonance (SPR) (Supplementary Figure S4A). The *K*_D_ for nsp10/16 interaction with immobilized nsp14wt was estimated at 6.04 ± 1.41 and 7.13 ± 0.84 μM in kinetic binding model and steady state, respectively, which is an order of magnitude higher than the *K*_D_ determined by MST. We attribute the difference to the immobilization constraint necessary in SPR and not present in MST. Despite the differences in *K*_D_ values, the SPR results show a similar trend to the results obtained using the MST; nsp10/16 binding to nsp14ExoN or to nsp14Δ is stronger than that of nsp10 alone (Supplementary Figure S2A). In fact, it was possible to saturate the nsp14 signal with nsp10/16 but not nsp10 alone.

To further define the heterotrimer complex and its components, we analyzed thermal denaturation profiles using nanoscale differential scanning fluorimetry (nanoDSF). Each of the individual system components (nsp10, nsp14wt and nsp16) was characterized by a single, distinctive denaturation temperature (Supplementary Figure S4B), indicating that structures of the functional domains within particular components collapse in a coordinated manner upon temperature increase. Nsp14wt was stabilized by complex formation with nsp10 (nsp10/14wt complex: *T*_m_ = 55.0°C), while nsp10 binding did not significantly affect the thermal stability of nsp16 (nsp10/16 complex: *T*_m_ = 45.5°C). The combination of nsp10, nsp16 and nsp14wt is characterized by a single sharp thermal denaturation peak with a distinctive melting temperature of 51.7°C, which further supports the heterotrimer formation.

### The nature of the nsp10–nsp16–nsp14 interactions

**Figure 4.**
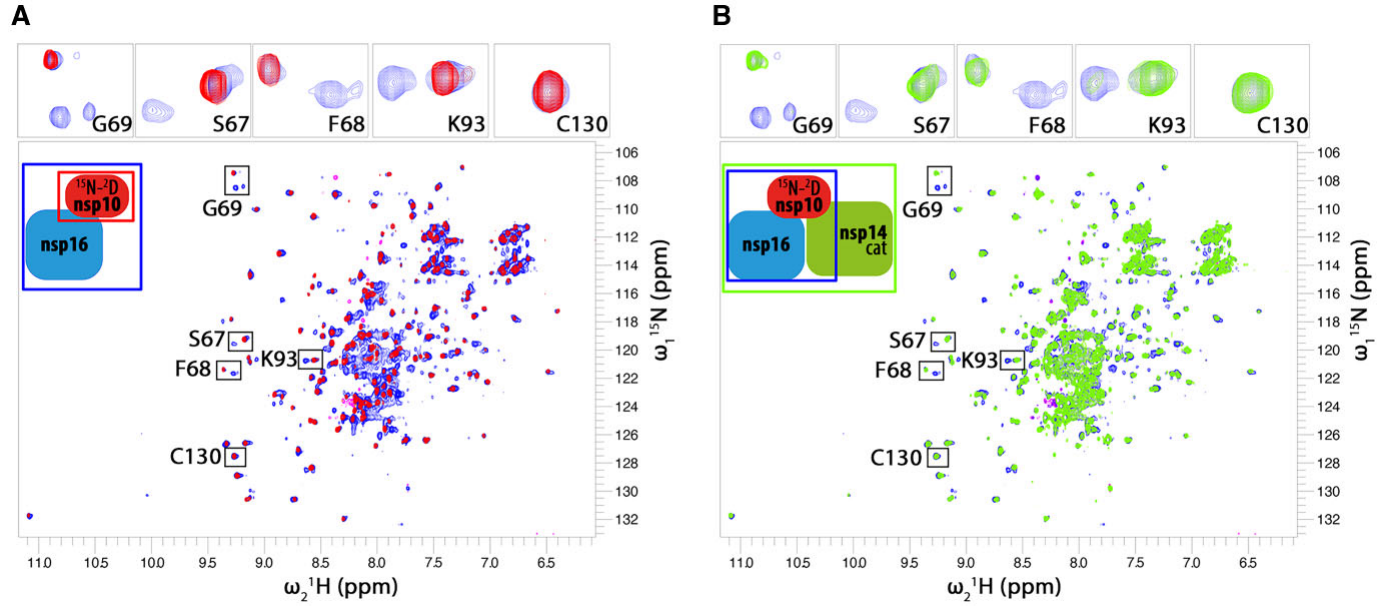
NMR titration of ^2^H^15^N-labeled nsp10 protein with unlabeled nsp16 and nsp14cat. Only resonances of the isotope-labeled nsp10 are observed in the NMR spectra. (**A**) The spectrum free nsp10 (100 μM; red peaks) is characteristic of a well-folded protein. The addition of approximately 200 μM of nsp16 (blue spectrum) causes the appearance of a second set of signals indicating the presence of free nsp10 and nsp10/16 in roughly equal amounts. Full saturation of nsp10 was not possible due to apo-nsp16 solubility limit. (**B**) To the nsp10/16 sample (blue), nsp14cat is added (green). The NMR signals of the free nsp10 population (the part of the blue spectrum that overlaps with the red one in panel (A) are not affected by the presence of nsp14cat (no change in free nsp10 amount and binding state is seen). The nsp10/16 complex population that appeared after nsp16 addition mostly disappeared in the presence of nsp14cat due to line broadening. This indicates that nsp14cat preferentially binds to the nsp10/16 heterodimer rather than the free nsp10. The inserts on top show magnified resonances marked on the whole spectrum for better readability.

In order to follow the interaction between the heterotrimer components in solution, we prepared ^2^H^15^N isotopically labeled nsp10 for nuclear magnetic resonance (NMR) analysis. The use of deuterated nsp10 and recording of transverse relaxation optimized spectroscopy (TROSY) heteronuclear correlation spectra (33) was required to reduce the linewidth of the NMR signals in the ternary complex. The other components of the heterotrimer were not labeled to avoid overcrowding of the spectra. The NMR spectrum of nsp10 shows well-dispersed resonances similar to the published spectra of nsp10 (34) (Figure 4). The addition of a 2-fold molar excess of nsp16 caused significant spectral changes with the appearance of a second set of signals. The new signals exist together with the signals recorded for apo nsp10 and contain two subsets of peaks (see Figure 4A, inserts). The experiment shows that both apo- and nsp16-bound nsp10 exist in a solution in roughly similar amounts (Figure 4A). Despite nominal molar excess of nsp16, nsp10 is not saturated, likely because the effective concentration of nsp16 in the sample is lower compared to nominal concentration due to significant precipitation of nps16 at concentrations required for NMR. The nsp10–nsp16 complex shows some structural heterogeneity as indicated by the presence of two subsets of signals. The presence of two sets of NMR signals for the complex indicates ‘slow exchange’ binding kinetics (35), a case when NMR experiment observes two states as separate populations of resonances due to ‘slow’, exchange between states (in order of tens of milliseconds to seconds). Next, we added a small molar excess of nsp14cat to the sample (Figure 4B). Interestingly, the population of apo-nsp10 signals was not affected by the presence of nsp14cat. However, the signals of the nsp10/16 species show substantial line-broadening. The line-broadening of the nsp10/16 population likely results from a combination of increased molecular weight and binding kinetics in the so-called NMR ‘intermediate exchange regime’. The lack of changes in the free nsp10 pool indicates that the presence of nsp14cat does not affect the apo-nsp10 to nsp10/16 balance, but only reflects binding to the nsp10/16 population. Interestingly, no interaction between the free nsp10 population and nsp14cat was observed in this experiment. This is due to the weaker affinity of nsp14 toward nsp10, compared to nsp16’s affinity to nsp10, and is in agreement with our MST (Figure 3D and Supplementary Figure S2) and SPR (Supplementary Figure S4A) data. The existence of the heterotrimeric complex in solution is thus preferred over the nsp10/14cat heterodimer. The excess of nsp14 does not cause noticeable alteration of apo-nsp10 resonances, this is similar to minimal spectra alteration upon nsp10-nsp14 binary titration.

### Functional consequences of nsp10/16/14wt heterotrimer complex formation

**Figure 5.**
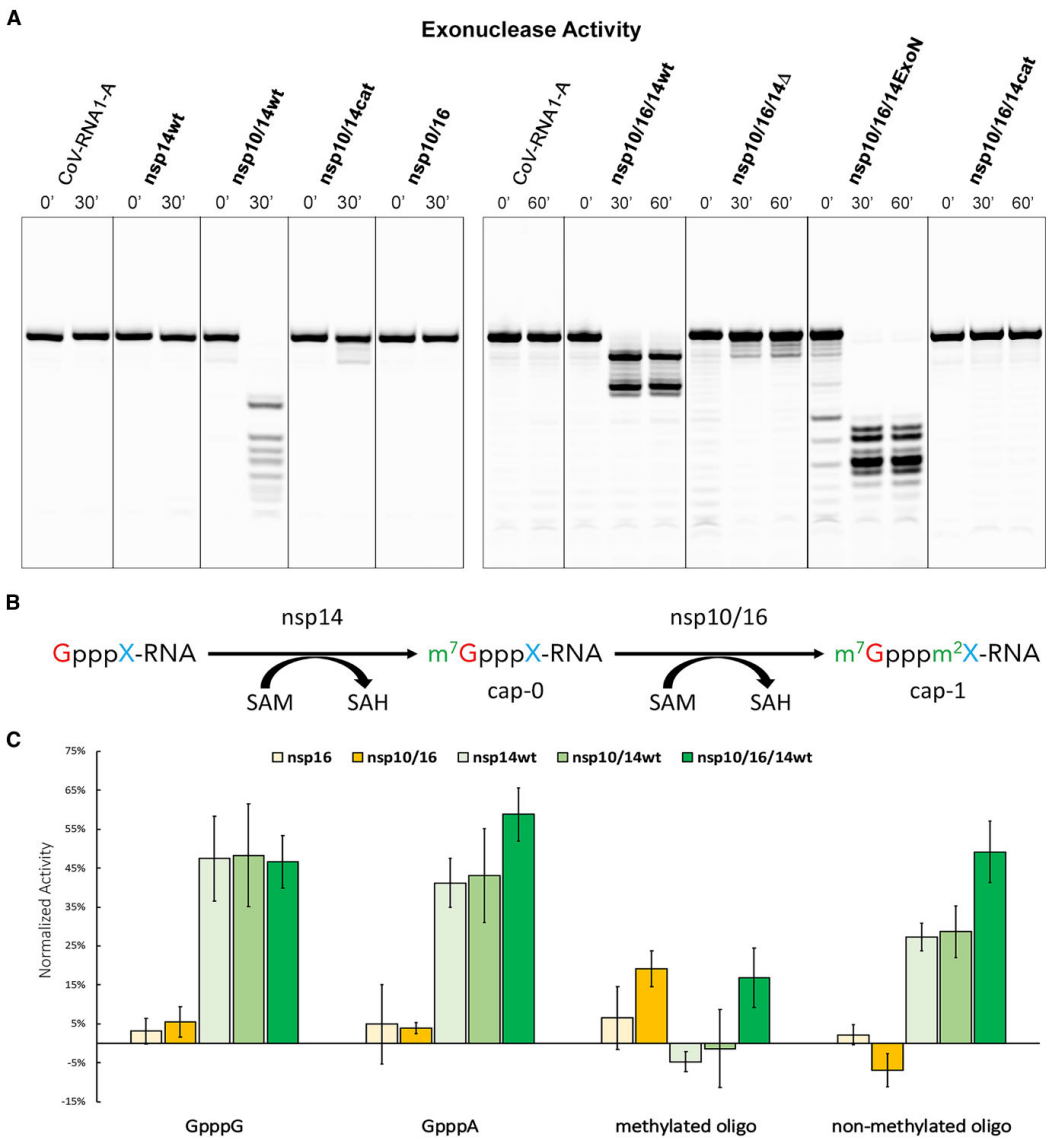
The formation of nsp10/16/14wt heterotrimer modulates the ribonuclease, but not methyltransferase activities. (**A**) Exonuclease activity. CoV-RNA1-A degradation profiles of nsp14wt, nsp10/14 (wt, cat), nsp10/16, with 0- and 30-min incubation, and profiles of nsp10/16/14 (wt, Δ, ExoN, and cat) from 0-, 30- and 60-min incubation. Merged from Supplementary Figure S5A–C. (**B**) Schematic view of mRNA methylation. X represents a nascent nucleotide (adenine (A) or guanine (G)). m^7^ represents methylation of the first guanine at position N7 by nsp14. m^2^ indicates 2′-*O* methylation of the nascent mRNA nucleotide. (**C**) Methyltransferase activity of tested complexes. The results were normalized at SAH concentration. All experiments were performed in duplicate. Average values with error bars (SD) are shown. The Y-axis was normalized to the negative control in which the volume of the inhibitor was replaced with buffer supplemented with DMSO.

The exoribonuclease activity of the nsp14 exonuclease domain is essential for proofreading during the virus replication process. In the *in vitro* conditions, nsp14 exhibits high processivity and non-specifically degrades nucleic acids (18,36,37). The proofreading function, however, requires tight control of nsp14 activity *in vivo*. We assessed whether the heterotrimer formation affects the exoribonuclease activity of nsp14 using RNA substrates (Figure 5A and Supplementary Figure S5). As reported earlier, the exoribonuclease activity of nsp14wt was nsp10-dependent (38,39). The nsp10/14wt complex yielded a distinctive and time-stable RNA degradation profile (Figure 5A). Heterotrimer formation significantly affected the exoribonuclease activity of nsp14wt – the time-stable end-product of RNA substrate degradation was distinct from that obtained using nsp10/14wt, demonstrating that the heterotrimer modulates the specificity of nsp14 exonuclease. Moreover, the RNA degradation pattern did not change during longer incubation times excluding the possibility of partial digestion by coexisting nsp10/14wt and nsp10/16 complexes instead of the heterotrimer as lower nsp10/14wt concentration would produce the same RNA degradation profile but in a longer time. Neither tested complexes containing the catalytic residue mutation in the exoribonuclease domain of nsp14 (nsp14cat, nsp10/14cat, and nsp10/16/14cat) nor nsp10/16 exhibited any exoribonuclease activity with tested substrates (Figure 5A and Supplementary Figure S5) demonstrating that the exonuclease domain is solely responsible for the exoribonuclease activity of tested complexes. Interestingly, the time-stable degradation pattern obtained using nsp10/14ExoN was distinct from that observed when the substrate was treated with nsp10/14wt demonstrating the modulation of the exoribonuclease activity by the presence of the nsp14 methyltransferase domain (Figure 5A and Supplementary Figure S5). The time-stable substrate degradation pattern obtained by incubation with nsp10/16/14ExoN was distinct from that obtained with nsp10/16/14wt, again demonstrating the modulating effect of the exonuclease activity by the methyltransferase domain of nsp14. Finally, the time-stable substrate degradation pattern obtained with nsp10/14ExoN was distinct from that obtained with nsp10/16/14ExoN once more exhibiting the modulatory effect of the heterotrimer complex on the exonuclease activity of nsp14 (Figure 5A and Supplementary Figure S5B–D).

SARS-CoV-2 nsp14 N7-methyltransferase domain transfers a methyl moiety from *S*-adenosylmethionine (SAM) to a substrate RNA, yielding N^7^MeGpppA/G product called cap-0, the RNA modification that is essential for efficient RNA translation and enhanced RNA stability (40); sequentially, cap-0 is a substrate for nsp16 O2-methyltransferase, which further methylates it to the mature cap-1 that is important in evading the cellular innate immune response (Figure 5B). Here, we tested whether the heterotrimer complex formation affects the nsp14-catalyzed N7 methylation. To follow the N7-methyltransferase activity of nsp14, we used an indirect assay to monitor changes in the level of a second reaction by-product, *S*-adenosylhomocysteine (SAH), using Homogeneous Time-Resolved Fluorescence (HTRF). No activity was detected in the absence of one of the substrates (SAM or RNA) or the tested enzyme (negative controls) validating the experimental setup. Also, sinefungin - a pan-methyltransferase inhibitor, halted the methyltransferase activities of all proteins reported here (41) (Supplementary Figure S6). Nsp14wt showed no preference for the nascent nucleotide, methylating both GpppG and GpppA, as demonstrated by the production of equal levels of SAH when using either nucleotide as a substrate (Figure 5C). The binary complex of nsp10/14wt and the ternary complex N7-methyltransferase activities were comparable to that of nsp14wt alone, indicating that binding to nsp10 or the heterotrimer complex formation had no noteworthy influence on the N7-methyltransferase activity of nsp14. N7-methyltransferase activity was also not seen for the already N7-methylated substrate (methylated oligo in Figure 5C).

Nsp16, presenting 2′-*O*-methyltransferase activity in the complex with its obligatory partner nsp10, shows no signal on the non-methylated substrates GpppA and GpppG, and only moderate activity on the methylated substrate (Figure 5C). As observed with N7-methyltransferase activity, 2′-*O*-methyltransferase activity is not affected by the heterotrimer complex formation.

We have also synthesized an in-house non-methylated substrate that can be first processed by SARS-CoV-2 nsp14 and later nsp10/16, yielding two SAH molecules. We observed no activity of nsp16 or nsp10/16 as the substrate lacks N7-methylation, a moderate activity for both nsp14 and nsp10/14 and almost twice as high signal for heterotrimer indicating sequential actions of both nsp14 and nsp16.

The nsp10/16/14Δ heterotrimer exhibits no exoribonuclease activity (see details in the Functional consequences of nsp10/16/14wt heterotrimer complex formation section), consistent with the previously reported importance of lysine at position 9 from the lid region in the exoribonuclease activity of the nsp10/14 complex (38). The importance of the lid in the exoribonucleolytic activity of nsp14 suggests the expected mechanism of a modulatory effect of the ternary complex on RNA substrate degradation by nsp14 (38).

The above data suggest that heterotrimer formation modulates the exonuclease activity of nsp14 but has no significant effect on the methyltransferase activities of nsp14 and nsp16. This is consistent with the fact that the methyltransferase domain of nsp14 is dispensable in nsp10 interaction since the nsp14ExoN binds nsp10 with *K*_D_ of 4.55 ± 2.68 μM; (Supplementary Figure S2) comparable to *K*_D_ of 2.40 ± 0.20 μM characterizing the interaction of nsp14wt with nsp10. The methyltransferase domain is also not predominantly involved in the heterotrimer formation, as the binding of nsp14ExoN and nsp10/16 is characterized by a *K*_D_ of 0.11 ± 0.13 μM, compared to the *K*_D_ of 0.28 ± 0.01 μM characterizing the interaction of nsp14wt and nsp10/16. The methyltransferase domain modulates the exonuclease specificity as demonstrated by different substrate degradation profiles (Figure 5A and Supplementary Figure S5). Further, by mutating the nsp14 exoribonuclease active site (nsp14cat), we tested whether the exoribonuclease activity of nsp14 affects its methyltransferase activity. No difference in N7-methyltransferase activity was observed between nsp14wt and nsp14cat, suggesting that the two activities are decoupled (Supplementary Figure S6).

### Structural characterization of the heterotrimer complex

The structure of the nsp10/16/14 heterotrimer was evaluated by Small Angle X-ray Scattering (SAXS). The overlaid chromatograms from SEC indicate a super-shift between the analyzed samples that follows theoretical molecular weights (Supplementary Figure S7A). The retention time in SAXS coupled with SEC correlated with the theoretical molecular weight assuming 1:1:1 molar ratio (Supplementary Figure S7B). Guinier analysis of scattering profiles established that the radii of gyration (Rg) of the heterotrimer complex at 40.3 ± 1.0 Å. Nsp14wt and the nsp10/14wt complexes were distinguished by significantly smaller Rgs (28.0 ± 0.5 and 30.1 ± 1.8 Å, respectively); the nsp10/16 complex was characterized by Rg of 21.0 ± 1.5 Å (Supplementary Table S3). These data correspond well with expected molecular weights of tested complexes at 1:1(:1) stoichiometry.

When reconstructed in real space using the indirect Fourier transform (GNOM software), the scattering profiles present roughly Gaussian shapes with significant tailing for nsp10/16/14wt and nsp10/14wt, indicating an elongated nature for the protein complexes, with peaks overlapping with radii of gyration obtained using Guinier analysis (Supplementary Figure S7C). Calculated maximal distances within scatterers support the trend established above, with nsp10/16 constituting the smallest complex at 80.0 Å, nsp14 at 95.8 Å, nsp10/14wt at 122.0 Å, and nsp10/16/14wt at 140.0 Å (longest axis).

**Figure 6.**
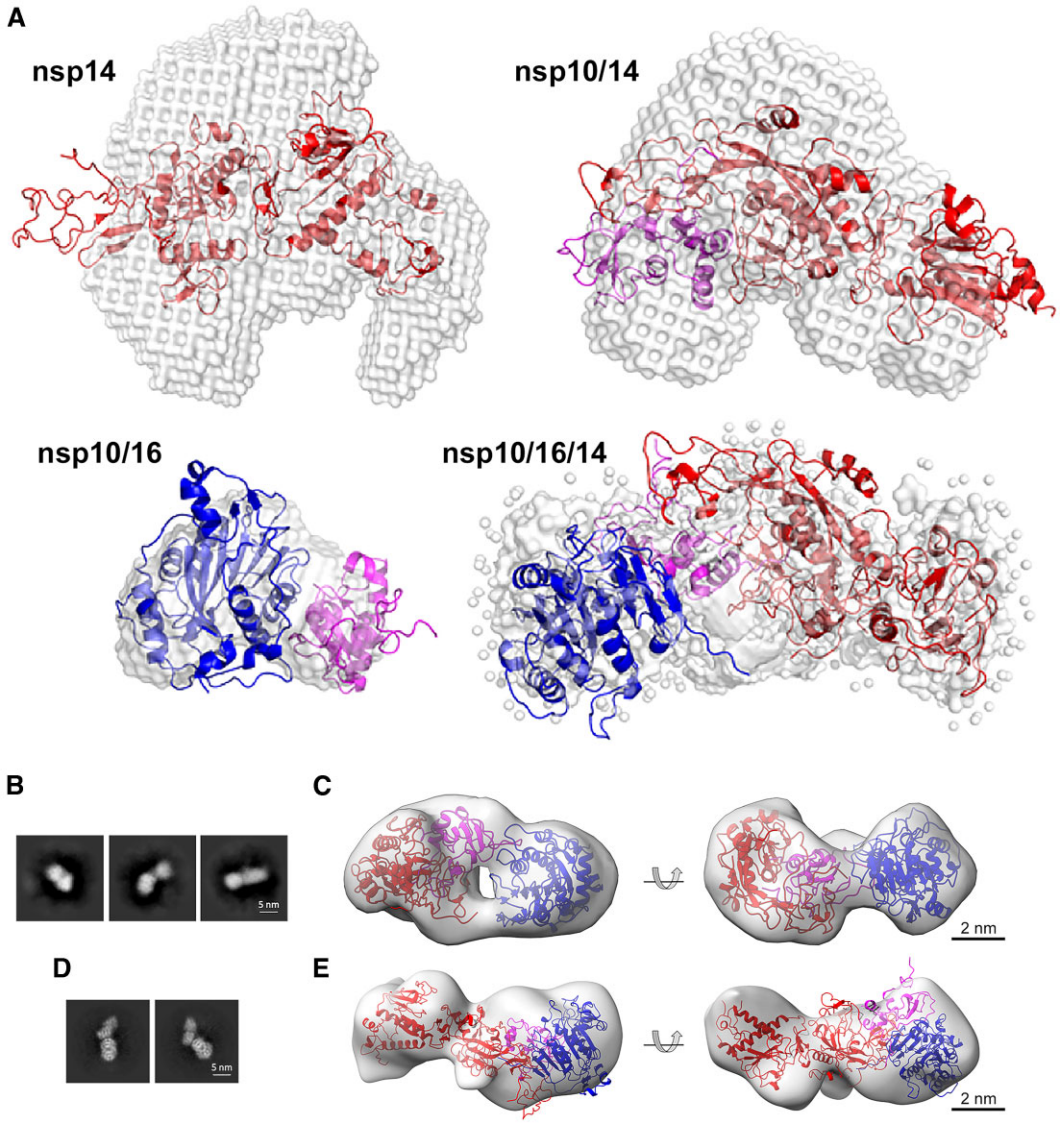
Heterotrimer structural characterization. (**A**) Molecular envelopes representing the experimental SAXS scattering profiles of nsp14wt (PDB ID: 7R2V), nsp10/14wt (PDB ID: 5C8U), nsp10/16 (PDB ID: 6W4H), nsp10/16/14wt (model generated using SREFLEX software). Overlaid are the best fits of crystallographic/theoretical models of relevant complexes. Color coding: nsp10 in magenta, nsp14 in red, nsp16 in blue, molecular envelopes in gray. (**B**) Transmission electron microscopy (TEM) characterization of the nsp10/16/14ExoN complex. Representative 2D classes obtained by template-free 2D classification of particles picked from NS-TEM micrographs. (**C**) Rigid body fit of SAXS-derived structure of heterotrimer complex into NS-TEM-derived 3D reconstitution map. Nsp14ExoN, nsp10, and nsp16 are represented as red, magenta, and blue ribbon models, respectively. The 3D reconstitution map is shown as transparent gray surface. (**D**) 2D classes obtained from particles from cryo-EM and the corresponding 3D volume (**E**). The size and shape of *ab initio* reconstituted density agrees with the expected heterotrimer structure. Significant dynamics of the complex prevented higher resolution analysis.

Molecular envelopes, which are consistent with the scattering profiles, were calculated using DAMMIF software (42) (Figure 6A). Crystal structures of nsp10/16, nsp14wt and nsp10/14wt and a model of the nsp10/16/14wt heterotrimer complex from our MD simulation, created assuming the lid hypothesis, were fitted into the envelopes using the SUPCOMB software. The crystal structures of nsp14wt and nsp10/14wt fit the molecular envelopes poorly, suggesting significant flexibility in solution (Figure 6A). The structure of nsp10/16 fills the envelope tightly suggesting a more rigid structure in solution. The initial model of the heterotrimer complex already filled the envelope relatively well and was further optimized via normal mode analysis using the SREFLEX software (43). The resulting heterotrimer model was characterized by a χ^2^ value of 1.08 for goodness-of-fit to the experimental SAXS data. The model fits the envelope tightly, suggesting the rigidification of nsp14 structure upon the formation of the heterotrimer complex (compared to nsp10/14). The decomposition of the nsp10/16/14wt scattering profile into volume fractions calculated from the crystal structures of the binary complexes nsp10/14, and nsp10/16, or the extracted individual proteins in the software Oligomer suggest that the signal can be divided 1:1 into nsp10/16 and nsp14 (Supplementary Table S3). This further implies that the nsp10/16 interface within the nsp10/16/14wt is retained, and it is nsp14wt that undergoes structural rearrangements upon heterotrimer complex formation.

The heterotrimer was further analyzed by transmission electron microscopy. Negatively stained samples of the nsp10/16/14wt complex formed from full-length components yielded a non-homogenous particle distribution, which precluded structural analysis. This indicates that the exonuclease and methyltransferase domains are flexibly connected in the heterotrimer. When the nsp14 methyltransferase domain was truncated, the heterotrimer consisting of nsp10/16/14ExoN yielded a more homogenous distribution allowing convergent classification (Figure 6B) and structural analysis. The reconstruction obtained from negative-stained transmission electron micrographs at 20 Å resolution shows elongated particles with approximate dimensions of ∼10 × 5 × 4.5 nm (Figure 6C). Necking is evident in the center of the particles, indicating that two larger structural components are connected by a component of lower molecular weight. The SAXS-derived structural model of the heterotrimer complex fits the experimental negative-stain transmission electron microscopy (NS-TEM) map reasonably well with CCmask/CCbox of 0.59/0.80. Nsp14ExoN and nsp16 fit the two globular regions of the map, connected by a region with a thinner density corresponding to nsp10. The central cavity suggested by SAXS data is visible in the electron microscope-derived map, indicating an overall match between the 3D reconstitutions obtained using each method.

To elucidate the structural details of the heterotrimer at higher resolution, we employed cryo-electron microscopy (cryo-EM) (Figure 6D, E). Testing several nsp14 variants and multiple conditions documented a significant structural heterogeneity related to the dynamic nature of the complex structure and thus prohibiting us from obtaining a high-resolution 3D map. We were eventually successful in getting a reasonable density for nsp10/16/14Δ heterotrimer of 12.03 Å resolution at 0.143 FSC (Supplementary Figure S8, Supplementary Table S4). The *ab initio* reconstructed 3D density corresponds well with the expected size and shape of the heterotrimer. Heterotrimers composed of other tested variants of nsp14 exhibited even higher structural heterogeneity.

Overall, the low-resolution structural data confirm the existence of the heterotrimer in solution. Together with the biochemical data, we conclude that nsp14 and nsp16 create a nsp10-centered heterotrimer, which modulates the activity of nsp14 exonuclease and enables optimal enzyme co-localization for methyltransferase activity.

## Discussion

It was demonstrated earlier that nsp10 serves as a protein co-factor of both nsp14 and nsp16 offering additional RNA binding sites and allosteric regulation (34). Since RNA methylation is a sequential process carried out by both nsp14 and nsp16, one might logically expect that nsp10 could bring nsp14 and nsp16 together into a heterotrimer complex. This should improve the kinetics and processivity of the system by spatial proximity of enzymes catalyzing consecutive reactions in the capping pathway. However, the crystal structures available until recently suggested otherwise. The binding interfaces of nsp14 and nsp16 overlap at the surface of nsp10, suggesting that the heterotrimer complex is not feasible without a major structural rearrangement of either nsp14 or nsp16 (Figure 1Ai).

Careful analysis of existing data demonstrates that the nsp10/16 interface relies on a solid network of hydrophobic interactions mediated by a rigid central antiparallel β1-sheet of nsp10; the helices α2, α3 and α4; a coiled-coil region connecting helix α1 and the sheet β1 and α helices 3, 4 and 10, as well as β-sheet 4 (Figure 1Aii). In particular, Val42 and Leu45 of nsp10 are embedded into hydrophobic pockets formed by helices α3, α4 and α10 of nsp16 to form typical protein-protein interaction interface.

Despite the fact that the interface between nsp10 and nsp14 buries a larger surface area compared to the nsp10/16 interface, the affinity characterizing the former complex is almost an order of magnitude weaker. This prompted us to speculate that part of the nsp10/14 interface does not significantly contribute to the affinity. The interactions within the N-terminal region of nsp14 involving primarily loops and other poorly structured regions, such as the N-terminal coil-coiled region that interacts with the α1' helix of nsp10, β-turn 1 and loops between β-sheet 2/3 and β-sheet 7/8 (Figure 1Aiii), seemed a probable candidate. Indeed, the nsp14 variant where 50 N-terminal residues are removed (nsp14Δ) retains nsp10 binding properties and is characterized by an affinity 2X stronger compared to wild-type nsp14, supporting the claim that the N-terminal region does not significantly contribute to the interaction. Interestingly, it is the N-terminal region of nsp14 which overlaps with the nsp16 binding site at the surface of nsp10. This allowed us to hypothesize that the heterotrimer complex is formed when nsp16 displaces the N-terminal region (lid) of nsp14 at the surface of nsp10 (Figure 1Biii). Indeed, nsp14wt, nsp14cat, nsp14ExoN and nsp14Δ readily form heterotrimer complexes with nsp16 and nsp10.

The lid hypothesis is supported by number of results provided in this work. The gyration radii and molecular weight of the heterotrimer complex derived from SEC-SAXS experiments are higher than the gyration radii and molecular weights of any of the components or binary complexes (Supplementary Figure S7). The molecular envelope derived *ab initio* from the scattering profile of the heterotrimer complex fits the model suggested by the lid hypothesis.

NS-TEM and cryo-EM reconstructions of 3D volume defining the nsp10/16/14ExoN heterotrimer complex accommodates the SAXS-derived model with high confidence, further supporting the lid hypothesis. NMR titration data clearly showed the preference of nsp14 to bind to nsp10/16 heterodimer and not nsp10 alone resulting in the heterotrimer formation, which is supported by our MST analysis (Figure 3D and Supplementary Figure S2) and by SPR (Supplementary Figure S4A). The large size of the heterotrimer made the direct NMR observation of components other than nsp10 difficult. Yet, the nsp10 readout clearly documents heterotrimeric interactions in the solution. The interactions were in slow and intermediate NMR-exchange regimes, suggesting nanomolar or low micromolar affinities, respectively, in agreement with affinities obtained by MST.

Overall, this study provides evidence that nsp14, nsp10 and nsp16 form a heterotrimer complex characterized by 1:1:1 stoichiometry, built around nsp10. The architecture of the complex follows the general arrangement previously observed in the nsp10/16 and nsp10/14 complexes, but nsp16 displaces the lid (N-terminal) of nsp14 at the nsp10 surface (Figure 1B). This allosteric rearrangement is feasible in the light of the lid flexibility observed previously by us (20). The heterotrimer complex brings together two consecutive activities required for RNA cap formation (nsp14-associated N7-methyltransferase and nsp16 2′-*O*-methyltransferase), likely contributing to the processivity of the RNA capping mechanism. The specificity of RNA exonuclease activity is determined by RNA interaction with all components of the complex as well as allosteric changes within the exonuclease domain itself (20). There are several point mutations in nsp14 reported that render SARS-CoV-2 inactive (44). Nsp10 and nsp16 bind to RNA too, therefore, they will modulate the specificity towards particular sequential and structural RNA motifs. The refolding of the lid to extend towards nsp16 in the heterotrimer is likely to occur, as hinted by the presence of a rigid fragment of the lid in the heterotrimer MD simulation and the high affinity of nsp14 towards the nsp10/16 complex. The presence of allosteric adaptation within nsp10, which improves affinity, presumably by freezing flexible elements and thereby reducing the entropic penalty of binding, is likely contributing to the effect, as high affinity is also observed for the nsp14Δ variant.

Nsp14 is likely in a dynamic equilibrium between its RNA-methylating heterotrimer state and being embedded within the RNA polymerase complex.

The nsp14-dependent proofreading mechanism allows the virus to reduce polymerase error frequency effectively, preventing the stall of the replication complex and generation of non-viable progeny (45). Beyond maintaining genome integrity, the ExoN domain is instrumental in facilitating the virus evasion of host immune responses by degrading the build-up of double-stranded RNA intermediates, byproducts of viral replication (46–49). While this action protects the infected cell from premature recognition by the host's immune system, exonuclease activity of nsp14 extends beyond just dsRNA substrates, and the unregulated activity may be detrimental, as shown by us and others (50). This underscores the importance of precise regulation of exonuclease activity to shield the viral RNAs, and while nsp10 has been previously identified as an activity modulator, this study shows that its function is complemented by the association with nsp16, facilitating effective viral RNA replication.

## Supplementary Material

gkae165_Supplemental_File

## Data Availability

The data that support the findings of this study are available from the corresponding author upon reasonable request. Synchrotron SAXS data from solutions of the SARS-CoV-2 proteins: SASDKT6 – SARS-CoV-2 non-structural protein 14 (nsp14); SASDKU6 – SARS-CoV-2 non-structural protein 10/non-structural protein 14 complex (nsp10/nsp14); SASDKV6 – SARS-CoV-2 non-structural protein 10/non-structural protein 16 complex (nsp10/nsp16); SASDKW6 – SARS-CoV-2 non-structural protein 10/non-structural protein 14/non-structural protein 16 triplex (nsp10/nsp14/nsp16).
