# Supplementary material for: Despite the odds: formation of the SARS-CoV-2 methylation complex
Source: Nucleic Acids Res. 2024 Mar 18;52(11):6441–58. doi: 10.1093/nar/gkae165 (PMC11194070; doi:10.1093/nar/gkae165)
Supplement: gkae165_Supplemental_File [file gkae165_supplemental_file.pdf]

Supplementary Table S1. Amino acid sequences of SARS-CoV-2 nsp10, nsp16, and nsp14 (wt, cat, ExoN, and Δ) proteins.

**nsp10**
MGSSHHHHHHSQDPENLYFQGAGNATEVPANSTVLSFCAFAVDAAKAYKDYLASGGQPITNCVK<br>MLCTHTGTGQAITVTPEANMDQESFGGASCCLYCRCHIDHPNPKGFCDLKGKYVQIPTTCANDPV<br>GFTLKNTVCTVCGMWKGYGCSCDQLREPMLQ
**nsp16**
MGSSHHHHHHSQDPENLYFQGSSQAWQPGVAMPNLYKMQRMLLEKCDLQNYGDSATLPKGIMM<br>NVAKYTQLCQYLNTLTLAVPYNMRVIHFGAGSDKGVAPGTAVLRQWLPTGTLLVDSDLNDFVSDA<br>DSTLIGDCATVHTANKWDLIISDMYDPKTKNVTKENDSKEGFFTYICGFIQQKLALGGSVAIKITEHS<br>WNADLYKLMGHFAWWTAFVTNVNASSSEAFLIGCNYLGKPREQIDGYVMHANYIFWRNTNPIQLS<br>SYSLFDMSKFPLKLRGTAVMSLKEGQINDMILSLLSKGRLIIRENNRVVISSDVLVNN
**nsp14wt**
MGSSHHHHHHSQDPENLYFQGMAENVTGLFKDCSKVITGLHPTQAPTHLSVDTKFKTEGLCVDIP<br>GIPKDMTYRRLISMMGFKMNYQVNGYPNMFITREEAIRHVRAWIGFDVEGCHATREAVGTNLPLQL<br>GFSTGVNLVAVPTGYVDTPNNTDFSRVSAKPPPGDQFKHLIPLMYKGLPWNVVRIKIVQMLSDTLK<br>NLSDRVVFVLWAHGFELTSMKYFVKIGPERTCCLCDRRATCFSTASDTYACWHHSIGFDYVYNPF<br>MIDVQQWGFTGNLQSNHDLYCQVHGNAHVASCDAIMTRCLAVHECFVKRVDWTIEYPIIGDELKIN<br>AACRKVQHMVVKAALLADKFPVLHDIGNPKAIKCVPQADVEWKFYDAQPCSDKAYKIEELFYSYAT<br>HSDKFTDGVCLFWNCNVDRYPANSIVCRFDTRVLSNLNLPGCDGGSLYVNKHAFHTPAFDKSAFV<br>NLKQLPFFYYSDSPCESHGKQVVSDIDYVPLKSATCITRCNLGGAVCRHHANEYRLYLDAYNMMIS<br>AGFSLWVYKQFDTYNLWNTFTRLQ
**nsp14cat**
MGSSHHHHHHSQDPENLYFQGMAENVTGLFKDCSKVITGLHPTQAPTHLSVDTKFKTEGLCVDIP<br>GIPKDMTYRRLISMMGFKMNYQVNGYPNMFITREEAIRHVRAWIGFAVAGCHATREAVGTNLPLQL<br>GFSTGVNLVAVPTGYVDTPNNTDFSRVSAKPPPGDQFKHLIPLMYKGLPWNVVRIKIVQMLSDTLK<br>NLSDRVVFVLWAHGFELTSMKYFVKIGPERTCCLCDRRATCFSTASDTYACWHHSIGFDYVYNPF<br>MIDVQQWGFTGNLQSNHDLYCQVHGNAHVASCDAIMTRCLAVHECFVKRVDWTIEYPIIGDELKIN<br>AACRKVQHMVVKAALLADKFPVLHDIGNPKAIKCVPQADVEWKFYDAQPCSDKAYKIEELFYSYAT<br>HSDKFTDGVCLFWNCNVDRYPANSIVCRFDTRVLSNLNLPGCDGGSLYVNKHAFHTPAFDKSAFV<br>NLKQLPFFYYSDSPCESHGKQVVSDIDYVPLKSATCITRCNLGGAVCRHHANEYRLYLDAYNMMIS<br>AGFSLWVYKQFDTYNLWNTFTRLQ
**nsp14ExoN**
MGSSHHHHHHSQDPENLYFQGMAENVTGLFKDCSKVITGLHPTQAPTHLSVDTKFKTEGLCVDIP<br>GIPKDMTYRRLISMMGFKMNYQVNGYPNMFITREEAIRHVRAWIGFAVAGCHATREAVGTNLPLQL<br>GFSTGVNLVAVPTGYVDTPNNTDFSRVSAKPPPGDQFKHLIPLMYKGLPWNVVRIKIVQMLSDTLK<br>NLSDRVVFVLWAHGFELTSMKYFVKIGPERTCCLCDRRATCFSTASDTYACWHHSIGFDYVYNPF<br>MIDVQQWGFTGNLQSNHDLYCQVHGNAHVASCDAIMTRCLAVHECFVKR
**nsp14Δ**
MGSSHHHHHHSQDPENLYFQGMTYRRLISMMGFKMNYQVNGYPNMFITREEAIRHVRAWIGFDV<br>EGCHATREAVGTNLPLQLGFSTGVNLVAVPTGYVDTPNNTDFSRVSAKPPPGDQFKHLIPLMYKGL<br>PWNVVRIKIVQMLSDTLKNLSDRVVFVLWAHGFELTSMKYFVKIGPERTCCLCDRRATCFSTASDT<br>YACWHHSIGFDYVYNPFMIDVQQWGFTGNLQSNHDLYCQVHGNAHVASCDAIMTRCLAVHECFV<br>KRVDWTIEYPIIGDELKINAACRKVQHMVVKAALLADKFPVLHDIGNPKAIKCVPQADVEWKFYDAQ<br>PCSDKAYKIEELFYSYATHSDKFTDGVCLFWNCNVDRYPANSIVCRFDTRVLSNLNLPGCDGGSLY<br>VNKHAFHTPAFDKSAFVNLKQLPFFYYSDSPCESHGKQVVSDIDYVPLKSATCITRCNLGGAVCRH<br>HANEYRLYLDAYNMMISAGFSLWVYKQFDTYNLWNTFTRLQ

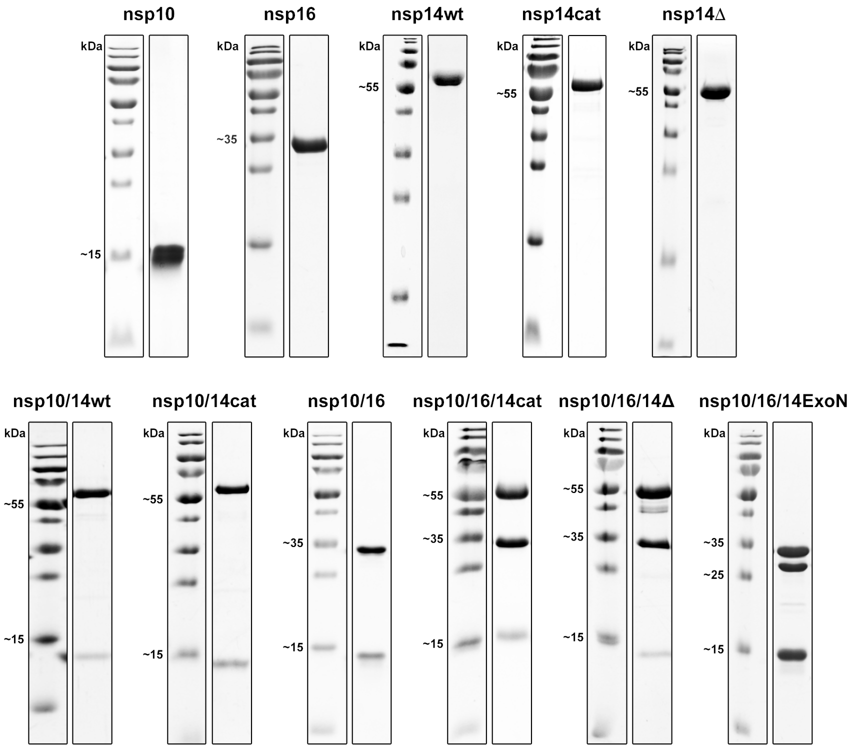

Supplementary Figure S1. SDS-PAGE illustrating the purity of the protein purifications from SEC. The top panel illustrates single proteins: nsp10, nsp16, nsp14wt, nsp14cat, and nsp14Δ. The lower panel shows protein complexes purified from their co-expression: nsp10/14wt, nsp10/14cat, nsp10/16, nsp10/16/14cat, nsp10/16/14Δ, and nsp10/16/14ExoN. For each protein sample, the corresponding protein ladder (ThermoFisher Scientific, 26616) is represented on the left, denoting the marker's molecular weight of interest.

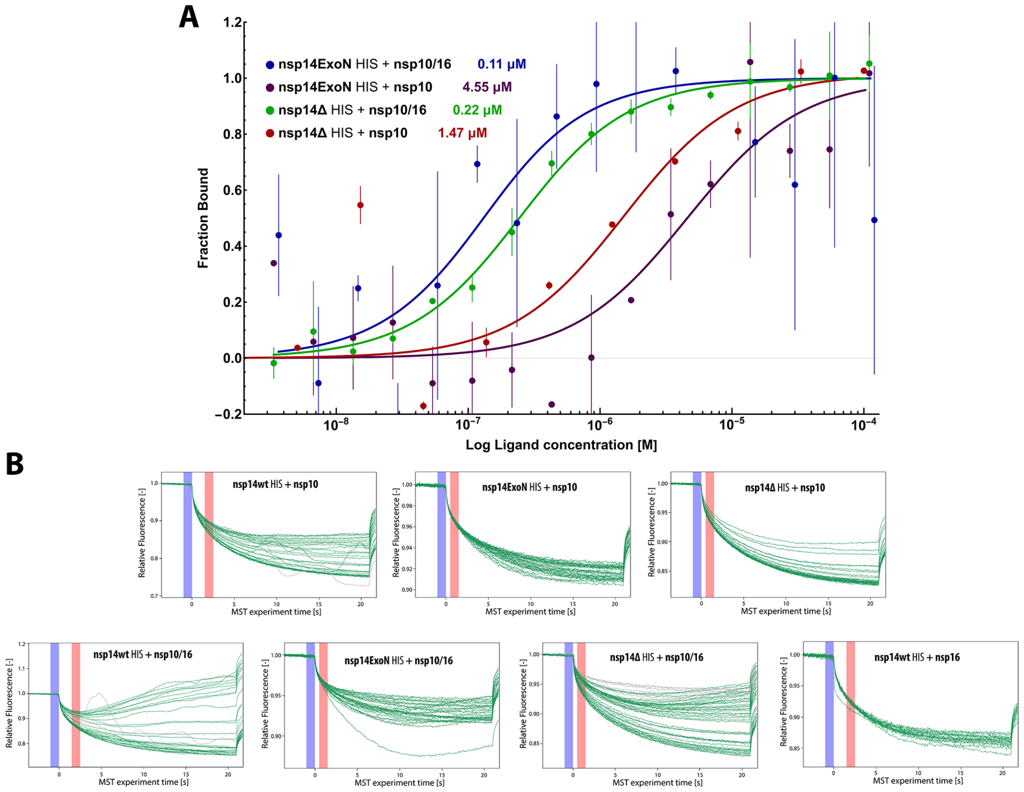

Supplementary Figure S2. MST analysis of nsp14ExoN and nsp14Δ affinities to nsp10 and nsp10/16. (**A**) The interaction between nsp14ExoN and nsp10/16 (blue) presented a K_D of 0.11 ± 0.13 μM; nsp14Δ and nsp10/16 (green) with K_D of 0.22 ± 0.03 μM; nsp14ExoN and nsp10 (purple) with K_D of 4.55 ± 2.68 μM; and between nsp14Δ and nsp10 (red) a K_D of 1.47 ± 0.42 μM. Experimental data are represented as mean (dots) with error bars. The binding model fits represented as solid lines. (**B**) Raw MST traces for the analysis of nsp14-HIS (wt, ExoN and Δ) interaction with nsp10, nsp10/16 and nsp16. The blue zone represents the cold region (equilibrium phase) and the red zone indicates the hot region (IR laser activation phase).

Analysis of the heterotrimer by RP-HPLC and mass spectrometry

The separation and quantitation of the nsp10, nsp14, and nsp16 proteins were done according to the methodology described in “Stoichiometry determination” (first paragraph), using Kinetex C18 column, which gave the best results out of the columns tested (including C4, C8, C18 columns). The resulting chromatogram for λ = 280 nm (**Supplementary Figure S3A**) and the map (y axis = λ [nm] / x axis = retention time [min] / z axis = peaks intensities [mA.U.]) obtained by the diode array detection (**Supplementary Figure S3B**) allowed a manual fraction collection. All the fractions underwent MS identification according to the procedure described in the article (“Stoichiometry determination” chapter, second paragraph).

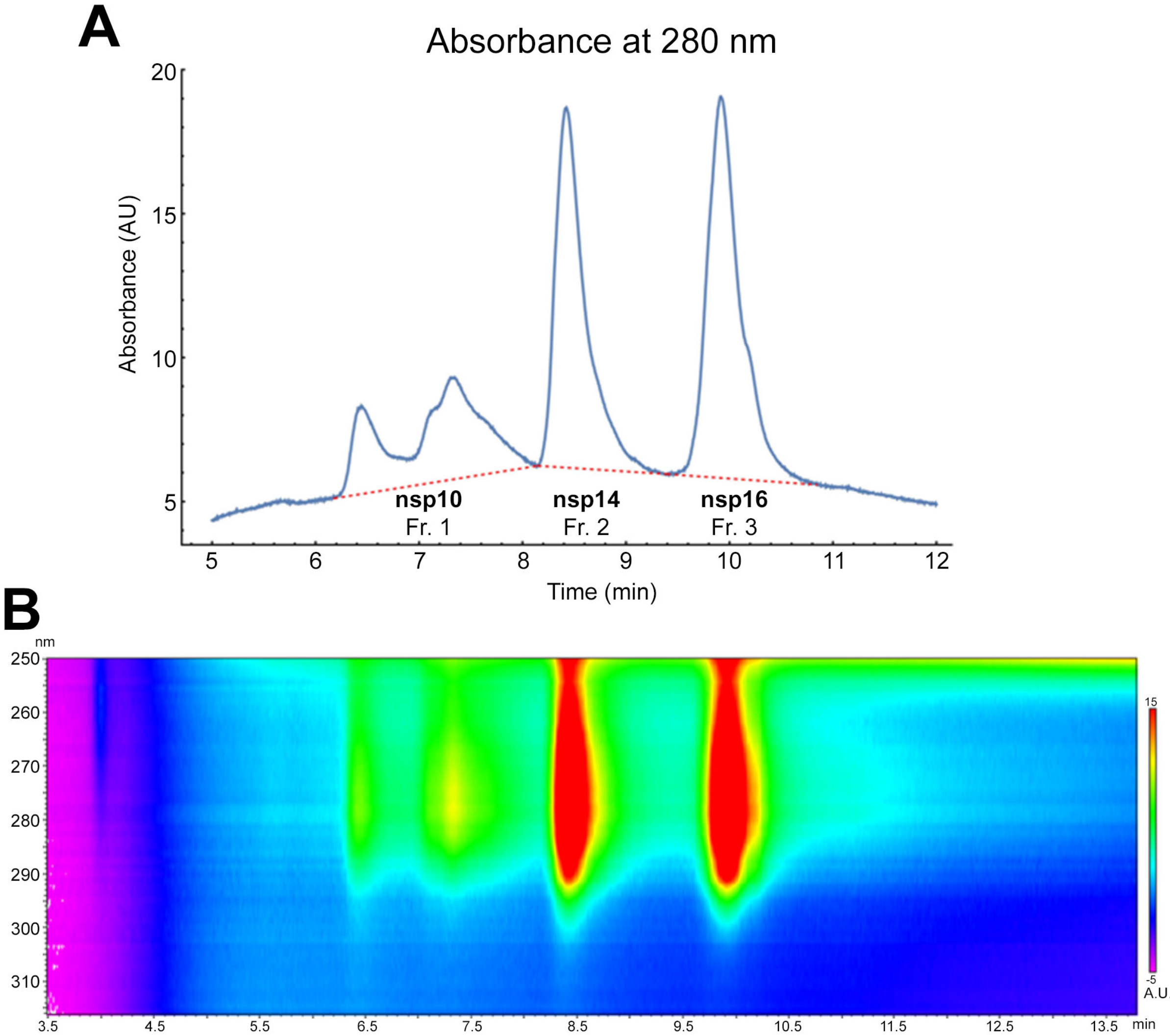

Supplementary Figure S3. Diode-array analysis of the heterotrimer. (**A**) The fractions acquired in the retention time ranges 6.3 - 8.0 min (Fr. 1), 8.2 - 9.3 min (Fr. 2) and 9.6 - 10.7 min (Fr. 3) slightly overlap. This overlap affects mainly nsp10, which is present in minor concentrations in Fr. 2 and Fr. 3 (see Supplementary Table 3). To check the level of cross-contamination among fractions, additional MS analyses were performed. The reasons for an atypical peak geometry noticed for nsp10 in the presented chromatogram remain unknown. The peak profile was very similar, independent of the column type, gradient applied, or chemical modifications of the sample compounds before the separation. (**B**) the diode array detection of the analyzed run. The heat map represents the intensity of the readouts at given wavelengths (y-axis) at a given elution times (x-axis) using the legend bar on the right side (high intensities are red, low intensities are blue).

Every acquired fraction underwent typical sample preparation for MS-based peptide mapping (freeze-drying, resuspension in the buffer, reduction, alkylation, trypsin digestion with following nanoLC-MS/MS analysis, see also “Stoichiometry determination” chapter, second paragraph). Protein identifications in each fraction revealed that Fr. 1 contains pure nsp10, Fr. 2 mainly nsp14 with minor contamination by nsp10, and Fr. 3 is composed mainly of nsp16 with minor contamination by nsp14 and nsp10. Such cross-contamination significantly influences the final ratio between proteins estimated by UV-VIS analysis only. To overcome this problem, at least 3 major peptides were identified from each protein during nanoLC. Next, the area under each chromatogram was calculated. The area for the same peptides from every MS analysis was added and averaged. The calculations led to the conclusion acquired in the table, that exemplary the distribution of nsp10 between the fractions is as follows: 64.5% in Fr. 1; 22 % in Fr. 2; and 13.5 % in Fr. 3 (data for nsp14 and nsp16 given in the table without further comment). Data normalization for diode array-based analysis (see **Supplementary Table S2**) is given in brackets: in Fr. 1 there was nsp10 detected only (100 in brackets), in Fr. 2 19.6 % of nsp10 and 80.4% of nsp14 (19.6 and 80.4 in brackets) and Fr. 3 10.9 % of nsp10, 8.2 % of nsp14 and 80.9 % of nsp16 (data for following fractions presented in the table without comment).

Supplementary Table S2. Analysis of the composition of the fractions indicated in the chromatogram from Supplementary Figure 2A. The column “average surface area” shows the areas under the peaks for corresponding proteins, taken directly from chromatographic separation (λ = 280 nm) after correction by the corresponding molar extinction coefficients. (chromatogram shown in the Supplementary Figure S3A). Column “total” presents virtual area values, additionally corrected with the aid of MS-based results of fractions composition analyses (the values shown in brackets in columns 2, 3 and 4. The virtual area values (column “Total”) were taken to final calculation of the ratio: nsp10:nsp14:nsp16 (columns: “percent” and "ratio”). Considering MS analyses: all were done with ion-trap constant ion accumulation time (max accu. time = 5 ms) to keep the peaks quantitation at the acceptable level.

	**Fr. 1** (fr. 6.3-8.0)	**Fr. 2** (fr. 8.2-9.3)	**Fr. 3** (fr. 9.6-10.7)	**Average surface area**	**Total**	**Percent**	**Ratio**
**nsp10**	64.5 % (100)<br>*62.0-67.0 %**	22 % (19.6)<br>*21.2-22.8%***	13.5 % (10.9)<br>*12.8-14.2 %****	145’756	205’562	38 %<br>*36.5-39.5 %*	**1.2**
**nsp14**	0 % (0.0)	90 % (80.4)<br>*86.8-93.2%***	10 % (8.2)<br>*9.3-10.7 %****	187’293	167’747	31 %<br>*29.6-32.4 %*	**1.0**
**nsp16**	0 % (0.0)	0 % (0.0)	100 % (80.9)<br>*94.5-105.5 %****	211’899	171’638	31 %<br>*29.3-32.7 %*	**1.0**

* average SD among tryptic peptide’s quantitation in fraction 1 (Fr. 1) was 5.03 % (extreme values: 2.9-12.6 %).
** average SD among tryptic peptide’s quantitation in fraction 2 (Fr. 2) was 7.23 % (extreme values: 6.0-9.5 %).
*** average SD among tryptic peptide’s quantitation in fraction 3 (Fr. 3) was 11.14 % (extreme values: 6.5-18.1 %).
NanoLC-MS analyses were done in 5 repeats for quantitation.

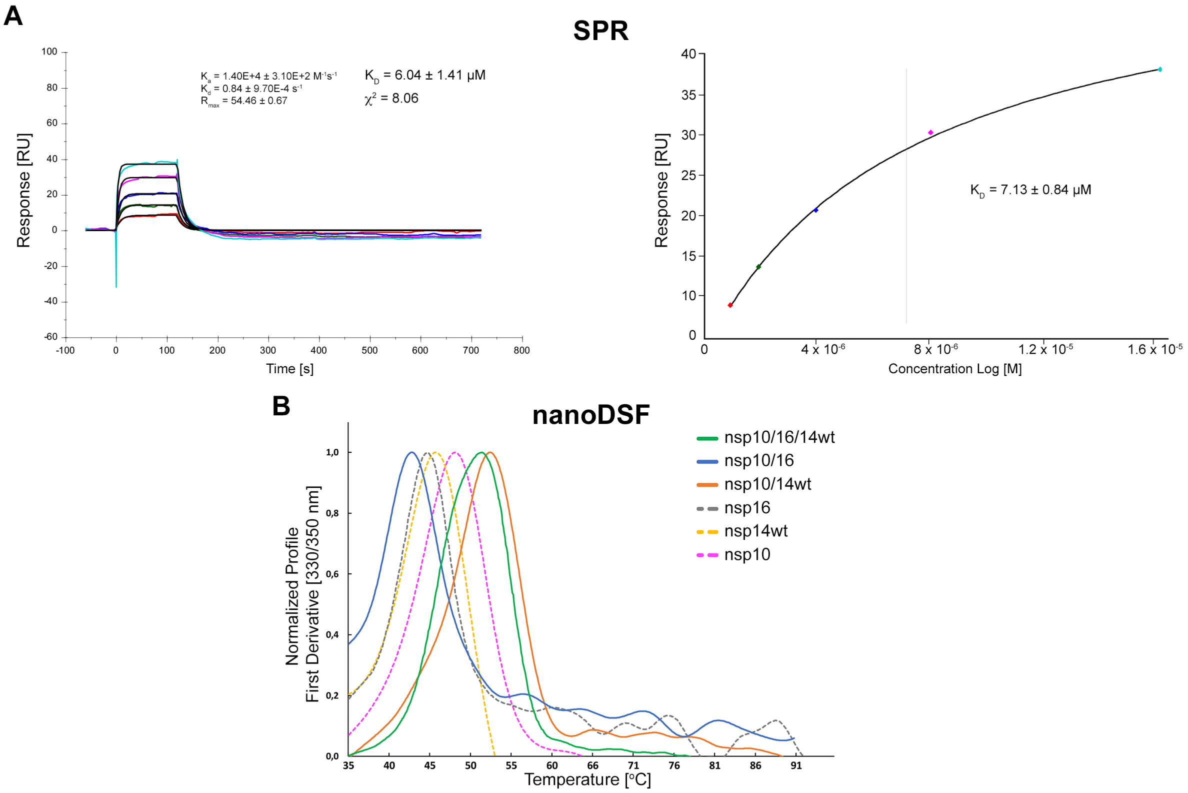

Supplementary Figure S4. (**A**) Surface Plasmon Resonance kinetic characterization of the interaction between immobilized nsp14cat and nsp10/16. Left panel: Sensograms of various response curves in RU versus time with increasing concentration of nsp10/16 in the solute (cycle 3 - 1 µM (red), cycle 4 - 2 µM (green), cycle 5 - 4 µM (blue) cycle 6 - 8 µM (pink), cycle 7 - 16 µM (cyan). The fitting to a 1:1 binding model is shown as black solid lines. Values of association constant K_a, dissociation constant K_d and resulting affinity K_D are shown in the graph. Right panel: Binding affinity curve for the immobilized nsp14cat interactions with increasing concentration of nsp10/16 fitted with a steady-state affinity model. (**B**) Melting profiles determined by nanoDSF for the nsp10/16/14wt heterotrimer complex (solid green); nsp10/16 (solid blue) and nsp10/14wt (solid orange) heterodimer complexes; and individual components nsp16 (dashed gray), nsp14wt (dashed yellow) and nsp10 (dashed turquoise).

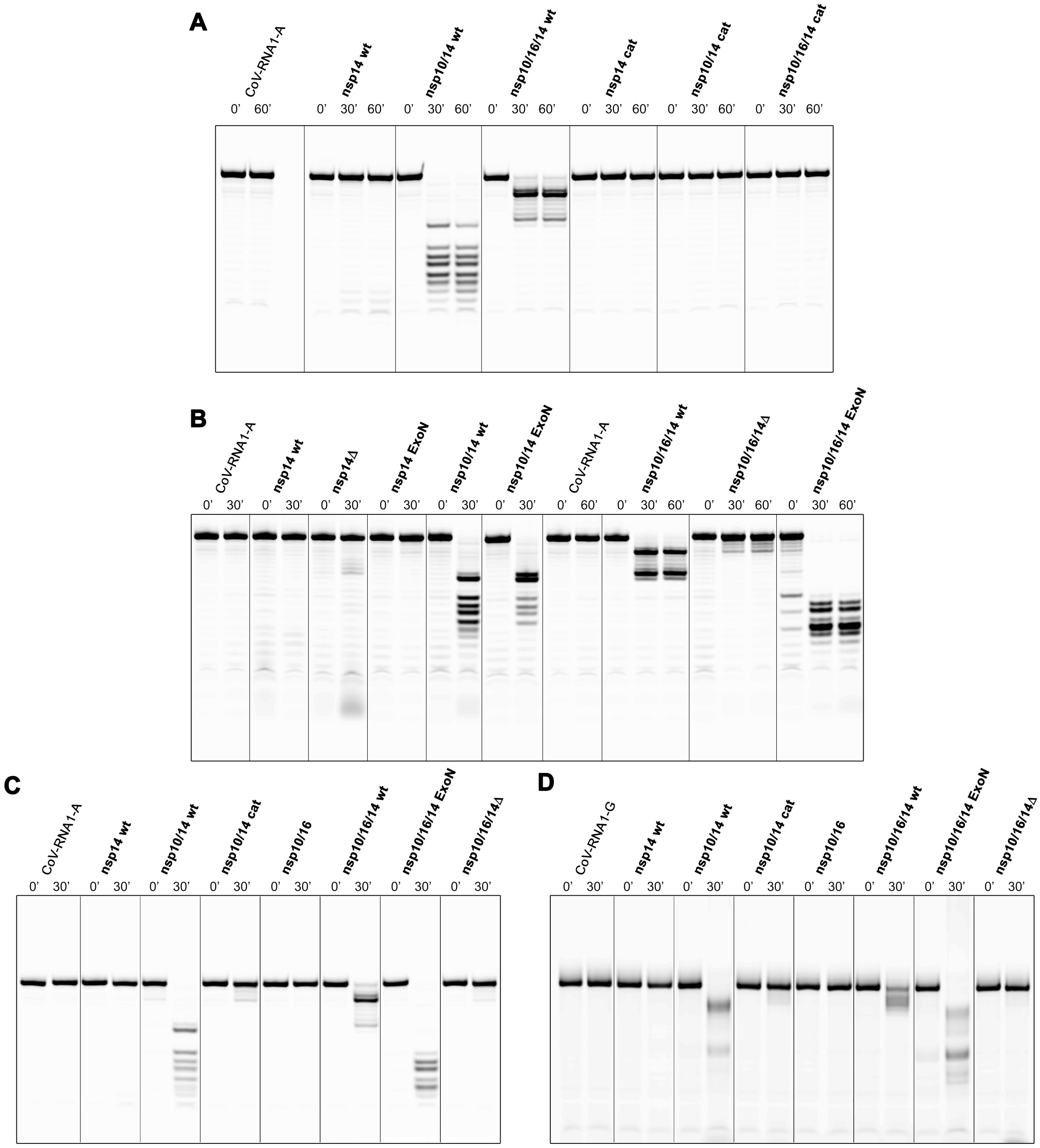

Supplementary Figure S5. Exonuclease activities. CoV-RNA1-A degradation profiles of single nsp14 (wt, cat, Δ and ExoN) and nsp10/14 heterodimers from 0 to 30 minutes, and from nsp10/14/16 heterotrimers with 0-, 30- and 60-minutes incubation. (**A-C**) full gels used to illustrate Figure 3A. Exonuclease activity assays with CoV-RNA1-A (**C**) and CoV-RNA1-G (**D**).

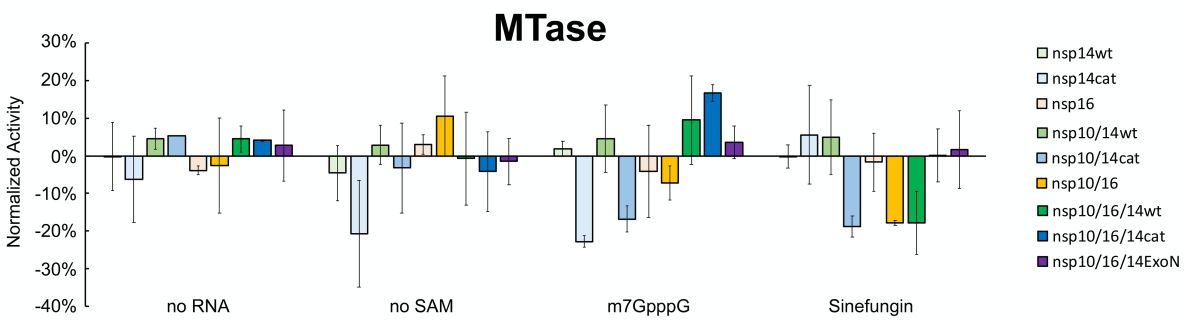

Supplementary Figure S6. Modulation of the methyltransferase activity by the protein partners. Methyltransferase negative controls. Methyltransferase activities for nsp14 (wt, cat), nsp10/14 (wt, cat), nsp16, nsp10/16, nsp10/16/14 (wt, cat and ExoN) without RNA, without SAM, with m7GpppG, and with sinefungin (50 µM).

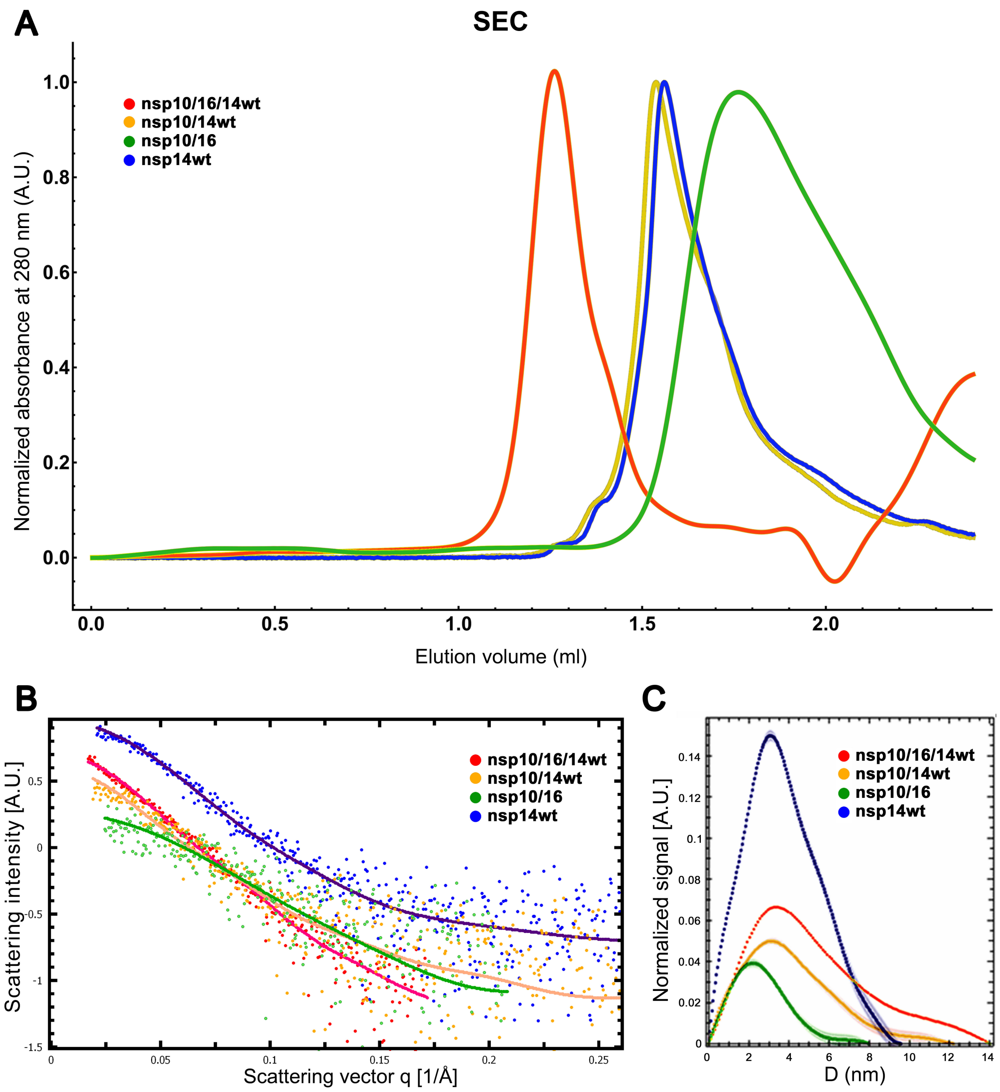

Supplementary Figure S7. SEC-SAXS. (**A**) Superimposed SEC chromatograms for nsp10/16/14wt (red), nsp10/14wt (yellow), nsp10/16 (green) and nsp14 (blue) directly before flow cell for SAXS measurement. The 280 nm absorption intensity was normalized for clarity. (**B**) SAXS scattering profiles resulting from merging the signal from SEC-SAXS experiments for nsp10/16/14 (red), nsp10/14 (yellow), nsp10/16 (green) and nsp14 (blue). The solid lines represent the fit of the experimental data to the real space models. In both cases, the position closer to the left (lower elution volumes or the curvature change at smaller scattering vector values q) indicates larger objects that were analyzed. Therefore, nsp10/14/16 represents the largest of the analyzed protein complexes, while nsp10/16 is the smallest, which is in agreement with their theoretical masses calculated from the amino acid sequences. (**C**) Real space reconstruction of SAXS profile (heterotrimer in red, nsp10/14 in yellow, nsp10/16 in green and nsp14 in blue.

Supplementary Table S3. Summary of the structural parameters derived from scattering profiles.

**a. Sample details**				
	nsp14wt	nsp10/14wt	nsp10/16	nsp10/16/14wt
Source organism	*Severe acute respiratory syndrome coronavirus 2*			
Source	*E. coli BL21*			
UniProt sequence ID (residues in construct)	P0DTD1 (5926-6452)	P0DTD1 (5926-6452)	-	P0DTD1 (5926-6452)
	-	P0DTC1 (4254-4392)	P0DTC1 (4254-4392)	P0DTC1 (4254-4392)
	-	-	P0DTD1 (6799-7096)	P0DTD1 (6799-7096)

Molecular weight from chemical composition (kDa)	59.9	74.7	48.5	108.5
SEC-SAXS column	AdvanceBio Bio SEC 300 column			
Loading concentration (mg/mL)	4	3.5	2.6	2.8
Injection volume (µL)	100			
Flow rate (mL/min)	0.16			
Running phase composition	50 mM Tris pH 8.5, 150 mM NaCl, 5 mM $MgCl_2$, 2 mM β-ME			
**b. SAXS data collection parameters**				
Instrument	BM29, ESRF, Grenoble France			
Detector	Pilatus2M			
Wavelength	0.99 Å			
Sample to detector distance	2.83 m			
**c. Structural parameters**				
	nsp14wt	nsp10/14wt	nsp10/16	nsp10/16/14wt
Guinier analysis				
I (0) (cm^{-1})	8.43 ± 0.13	3.22 ± 0.13	1.73 ± 0.09	5.26 ± 0.11
Rg(Å)	28.0 ± 0.5	30.1 ± 1.8	21.0 ± 1.5	40.3 ± 1.0
qRg(Å-1)	0.59 – 1.51	0.59 – 1.32	0.52 – 1.39	0.76 – 1.4
P(r) analysis				
Rg(Å)/I (0) (cm-1)	29.4/8.61	32.5/3.28	21.1/1.64	41.2/5.23
Theoretical Rg from high-resolution models	26.41	33.49	22.53	36.72
Dmax (Å)	95.8	122.0	80.0	140.0
Total quality estimate	0.94	0.78	0.75	0.77
Molecular weight estimate/predicted (kDa)	33.1/60	28.9/75	20.6/48.5	83.2/108.5
Oligomerization state	monomeric			
**d. Shape model-fitting results**				
DAMMIF (10 runs)	nsp14wt	nsp10/14wt	nsp10/16	nsp10/16/14wt
q_{max} range for fitting(Å-1)	0.26	0.26	0.21	0.17
Symmetry, anisotropy assumptions	P1, none			
NSD (standard deviation)	1.42 (0.07)	1.10 (0.07)	1.29 (0.14)	0.80 (0.08)
Chi-squared	1.16	1.09	1.07	1.08
Resolution (from SASRES) (Å)	43 ± 3	39.3 ± 3	33.3 ± 3	38.3 ± 3
SASDBD IDs	SASDKT6	SASDKU6	SASDKV6	SASDKW6
**e. Oligomer volume fractions**				

volume fractions χ^2 1.06				
nsp10/16 (6YZ1)	nsp16	nsp14wt	nsp10	nsp10/14wt (5CU8U)
53 %	0 %	47 %	0 %	0 %

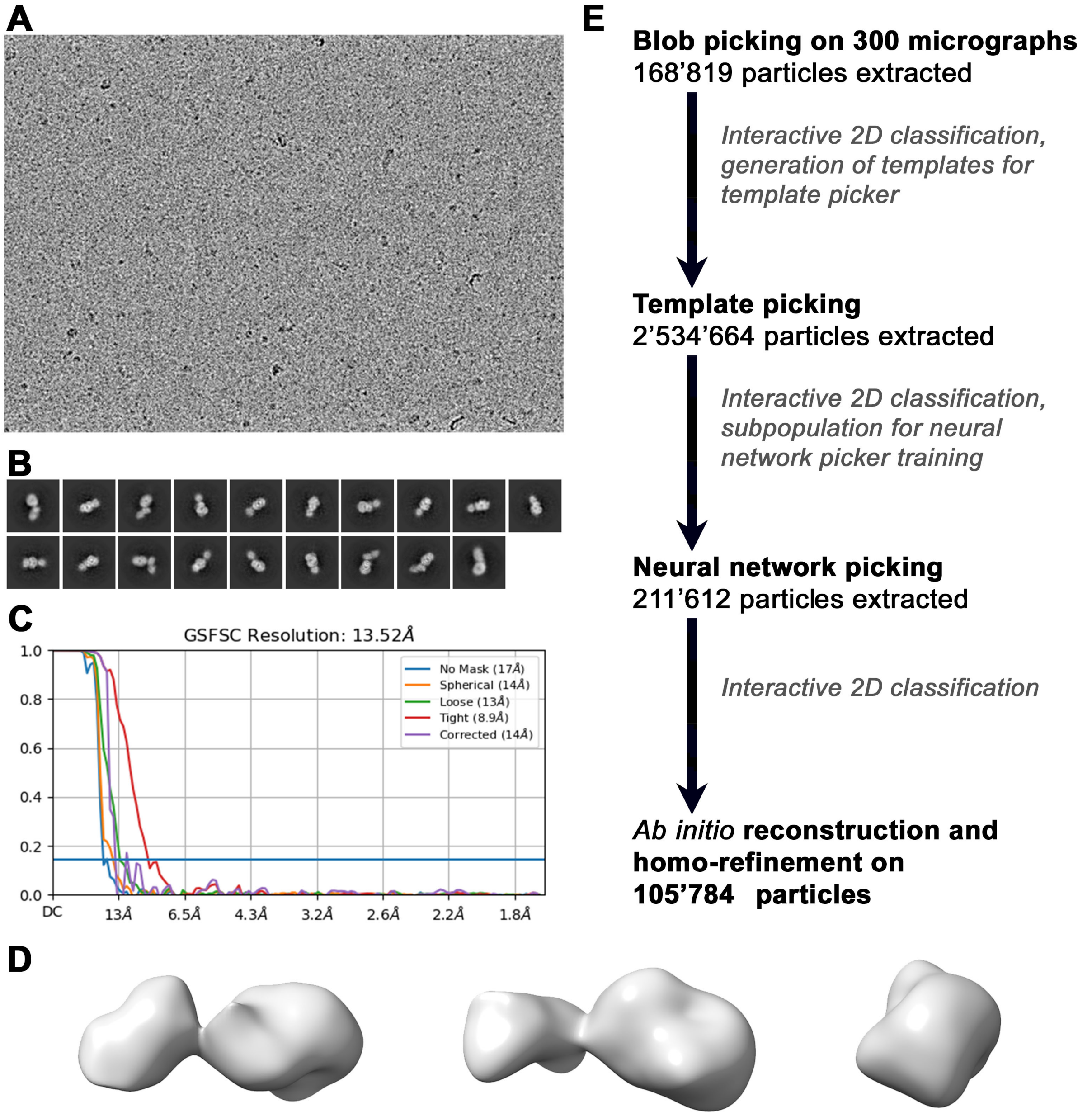

Supplementary Figure S8. cryoEM data processing summary. (**A**) Representative micrograph with particles in the ice. (**B**) Selected 2D classes used for reconstruction. (**C**) FSC half maps resolution determination. (**D**) 3D volume seen from different sides, each 90 degree rotated. Additionally, an overview of the cryo-EM single particle analysis is presented. (**E**) Overview of the cryoEM single particle analysis in cryoSparc.

Supplementary Table S4. Summary of the structural parameters derived from scattering profiles.

**Data acquisition**	Titan KriosG3i @ Solaris
High tension	300 kV
Spherical Aberration	2.7 mm
Pixel size	0.86 A
Total dose on vacuum	40.47 e/A^2
Frames per movie	40
defocus range set [µm]	-2.1, -1.8, -1.5, -1.2, -0.9
Stage tilt	0
Imaging mode	EFTEM
Magnification of whole image	11’500x

Magnification of exposure image	105’000x
Beam size	0.75 µm
C2 aperture	50 µm
Used filters	GatanBioQuantum energy filter
Energy slit	20 eV
Used mode	Gatan K3 counting mode
Dose rate on vacuum	15.83 e/px/s
Exposure image resolution	5’760 x 4’092
Physical pixel	Super Resolution off
EPU version	2.10.0.1941REL
**Data processing packages**	CryoSparc 4.0.0, Chimera 1.15, Namdinator, Phenix
Initial particle number	2’534’664
Final particle number	105’784
